# Comprehensive exome profiling identifies *ARHGEF12* mutation as a driver in gastric cancer with ovarian metastasis

**DOI:** 10.7150/thno.113382

**Published:** 2025-07-24

**Authors:** Mingda Zhang, Guoyu Chen, Xiaolin Lin, Yingwen Zhang, Longyu Shi, Shanshan Li, Yanxin Li, Xiuying Xiao, Haizhong Feng

**Affiliations:** 1 State Key Laboratory of Systems Medicine for Cancer, Ren Ji Hospital, Shanghai Cancer Institute, Shanghai Jiao Tong University School of Medicine, Shanghai, 200127, China.; 2 Department of Oncology, Ren Ji Hospital, Shanghai Jiao Tong University School of Medicine, Shanghai 200127, China.; 3 Pediatric Translational Medicine Institute, Department of Hematology & Oncology, Shanghai Children's Medical Center, Shanghai Jiao Tong University School of Medicine, National Health Committee Key Laboratory of Pediatric Hematology & Oncology, Shanghai, 200127, China.

**Keywords:** gastric cancer, ovarian metastasis, ARHGEF12, cancer-associated fibroblasts, exosomes

## Abstract

**Rationale:** Gastric cancer (GC) with ovarian metastasis (OM) represents a distinct subtype of peritoneal metastasis in female patients, characterized by limited therapeutic options and poor prognosis, with molecular features and mechanisms that remain unknown.

**Methods:** We performed whole-exome sequencing (WES) analysis of matched GC samples, with OM or peritoneal metastasis (PM), to identify mutational profiles that contribute to OM. We further validate these findings through in vitro and in vivo experiments.

**Results:** We characterized specific mutated genes in GC with OM, including *FLCN*, *DNAJC13*, *DSC3*, *SLC9A3*, *ADGRV1*, *SCAPER*, and *ARHGEF12*. Moreover, these genomic mutations are recurrent in both GC and ovarian cancer. We further identified the E620K mutation of *ARHGEF12*, a Rho guanine nucleotide exchange factor, as a novel risk locus in GC with OM. Ectopic expression of the E620K mutant increased cell migration, invasion and colony formation in vitro, as well as OM in animals bearing GC xenograft tumors. Mechanistically, *ARHGEF12* E620K mutation upregulated *ITGA6* expression through Rap1 signaling pathway activation and promoted tumor-derived ITGA6-high exosome formation, which were preferentially uptaken by ovarian fibroblasts. Reciprocally, ovarian fibroblasts educated by ITGA6*-*high exosomes exhibited cancer-associated fibroblasts (CAFs) phenotypes and enhanced tumor cell proliferation, thereby initiating the early stage of pre-metastatic niche formation.

**Conclusions:** Our study provides comprehensive clinical exome profiling, identifies *ARHGEF12* mutation as a new driver, and reveals that ITGA6 acts as an early predictive marker in GC with OM.

## Introduction

Gastric cancer (GC) is the fifth most common cancer and the fifth leading cause of cancer-related death worldwide 1. Ovarian metastasis (OM) of GC, also known as Krukenberg tumor, is a unique type of peritoneal metastasis (PM) in female patients, with a median survival time of 8-14 months 2, 3. Although the reported incidence of OM ranges from 0.3% to 6.7%, autopsy studies have revealed that the actual incidence may be between 33% and 41% 4, 5. Given the absence of comprehensive genomic profiling, the diagnosis and treatment of GC with OM remain challenging 6, 7.

Extensive genomic projects have adopted high-throughput technologies, including whole genome sequencing (WGS), whole-exome sequencing (WES) and targeted sequencing, to elaborate the genomic mutation landscape of GC 8-10. Several significant single-nucleotide variants (SNVs) and recurrent copy number alterations (CNAs) have been reported, making notable contributions to advancements in the categorization and understanding of GC 11, 12. Recently, a WES analysis of GC with OM after chemotherapy validated that *CLDN18* fusions enhance response to paclitaxel treatment 13. Furthermore, a single-cell RNA sequencing (scRNA-Seq) analysis of GC with OM or PM discovered ovarian fibroblasts express high levels of estrogen receptor (ER) 14. However, the genomic studies of GC with OM excluding chemotherapy remain unclear.

Rho GTPases are key regulators of cytoskeleton dynamics, playing critical roles in cell polarization and migration 15-17. The canonical Rho GTPases are regulated by guanine nucleotide exchange factors (GEFs). Rho Guanine Nucleotide Exchange Factor 12 (ARHGEF12), also named as Leukemia-associated Rho guanine nucleotide exchange factor (LARG), is a RHOA-specific GEF that plays a crucial role in various cellular processes 18. *ARHGEF12* is critical for cytokinesis, cell protrusions and retractions, neuroblastoma differentiation, and infectious synapse formation 17, 19-21. *ARHGEF12* mutations were initially identified in acute myeloid leukemia and B-cell lymphoma, *KMT2A-ARHGEF12* fusion caused hematologic disorders and associated with poor prognosis 22-24. However, the functions of *ARHGEF12* in GC remain unclear.

In this study, we enrolled fourteen GC patients, of which eight with OM and six with PM. All patients underwent gastrectomy and metastasectomy as primary treatment and all specimens were collected prior to chemotherapy, including primary tumors (PT), matched adjacent tissues (AT) and distant metastases. We performed WES to investigate molecular alterations associated with OM. By comparing the genomic mutations across these samples, we characterized several significantly mutated genes in GC with OM. We further identified the E620K mutation of *ARHGEF12* as a novel risk locus in GC with OM, as evidenced by RNA sequencing and functional studies.

## Materials and Methods

### Patients and samples

We obtained fresh-frozen primary tumors, with matched adjacent tissues and distant metastases for 14 gastric cancer (GC) patients who underwent gastrectomy and metastasectomy as primary treatment at Renji Hospital. We collected peripheral blood samples from 20 GC patients with ovarian metastasis (OM) and 20 patients with peritoneal metastasis (PM). The study protocol was approved by the Clinical Care and Use Committee of Ren Ji Hospital (Shanghai, China). Written informed consent was obtained from all participants in the study. DNA was extracted from these specimens using a QIAamp DNA Mini kit (QIAGEN). The clinical characteristics are shown in Table S1.

### Whole-exome sequencing (WES) analysis

WES was performed on the DNA of all samples by Illumina. Sequencing reads were aligned to the human genome reference (hg38) using the Burrows-Wheeler Aligner (BWA). PCR duplicates were identified using Picard MarkDuplicates. Read quality was normalized using the GATK base recalibration. Somatic mutation calling and filtering were done following the GATK best practices workflows. Briefly, the potential somatic short variations were called by GATK Mutect2, cross-sample contamination were estimated by GetPileupSummaries and CalculateContamination, orientation bias were learned by orientation bias. Then potential variations were filtered using GATK MuTect2. We further excluded mutations with low total read coverage (<10 reads). The filtered reads were functionally annotated by ANNOVAR.

### Functional enrichment analysis of gene sets

Enriched functions in the mutated gene sets were analyzed using the Gene Ontology (GO) categories and the Kyoto Encyclopedia of Genes and Genomes (KEGG) database in clusterProfiler 25.

### Cell lines and cell culture

Human gastric cancer cell lines HGC27, AGS, MKN45, MGC803, MGC823, SGC7901, a human gastric mucosal epithelial cell line GES, and the mouse gastric cancer cell line mouse forestomach carcinoma (MFC) were purchased from the Chinese National Infrastructure of Cell Line Resource (Beijing, China). All cell lines were cultured in RPMI-1640 (Corning) supplemented with 10% FBS (Gibco) and cultured in an incubator at 37 °C with 5% CO_2_.

### Plasmid and transfection assays

ARHGEF12 shRNAs and sgRNAs were generated as previously described 26. The ARHGEF12 shRNAs and sgRNAs sequences are listed in Table S2. The cloning of full-length ARHGEF12 was based on NCBI sequence NM_015303.3. Full-length ARHGEF12 was cloned into a pLVX-Puro vector (Clontech) for subsequent cloning. Different versions of ARHGEF12 (wild-type, G195R, E620K, G659R, S671P, P1314S, S1486T, A1532V) were cloned into a pLenti-HA-P2A-BSD vector (Addgene) and confirmed by DNA sequencing. ITGA6 and ITGB2 cDNAs were purchased from GeneChem (Shanghai, China). They were sequenced and subcloned into the pLVX-Puro vector (Clontech). Targeted DNAs and packaging plasmids were transfected into the HEK293T cells using the HieffTrans Liposomal Transfection Reagent (40802ES08, YEASEN) following the manufacturer's instruction. The supernatants were collected and filtered at 72 h after transfection. Targeted Cells were infected with 5 µg/ml polybrene (Sigma-Aldrich). Infected cells were selected with puromycin or blasticidin after infection. Multiple monoclonal cultures were screened for sgRNA by Western blotting and RT-PCR analyses.

### Antibodies and reagents

An antibody against ARHGEF12 (1:1000 for WB, 1:200 for IHC, #PA5-101847) was purchased from Invitrogen. Antibodies against α-tubulin (1:1000 for WB, #66031-1-Ig), GAPDH (1:1000 for WB, #60004-1-Ig), ITGA6 (1:1000 for WB, #27189-1-AP), ITGB2 (1:1000 for WB, #10554-1-AP), Alix (1:1000 for WB, #12422-1-AP), CD63 (1:1000 for WB, #25682-1-AP) were purchased from Proteintech. Antibodies against F4/80 (1:500 for IF, #30325), Talin-1 (1:1000 for WB, #4021), ERK (1:1000 for WB, #4695), Phospho-ERK (1:1000 for WB, #4370), RHOA (1:1000 for WB, #2117), PDGFRβ (1:1000 for WB, #3169), FAP (1:1000 for WB, #66562) were purchased from Cell Signaling Technology. Antibodies against α-SMA (1:1000 for WB, 1:500 for IF, #ab7817), F-actin (1:250 for IF, #ab130935), RAP1A (1:1000 for WB, #ab175329), TSG101 (1:1000 for WB, #ab125011) were purchased from Abcam. 5-FU, Oxaliplatin, Docetaxel and Estradiol were from Med Chem Express. Y16 was from Selleck Chemicals. Dil and Dio were from Beyotime.

### Immunohistochemistry (IHC) staining

Matched tumor specimens, adjacent tissues and ovarian metastases were prepared as 5 µm formalin-fixed, paraffin-embedded (FFPE) sections. Tissue sections were then incubated with primary antibodies against ARHGEF12. The proportion of ARHGEF12 staining was calculated as the percentage of positive tumor cells among all tumor cells. The intensity score was assigned for the average intensity of positive tumor cells (0, negative; 1, weak; 2, intermediate; 3, strong). H-score (ranging from 0 to 300) was calculated by multiplying the intensity score by the positive proportion. The sections were evaluated by three pathologists blinded to the nature of the samples.

### Immunofluorescence (IF) assay

Cells were fixed with 4% polyformaldehyde and permeabilized with 0.1% Triton X-100 for 10 minutes as previously described 27. After being blocked with 10% goat serum in PBS, cells were treated with F-actin, F4/80 and α-SMA. Primary antibodies were visualized by goat anti-mouse IgG bound to Alexa Fluor 647 (1:500, #ab150115, Abcam) and goat anti-mouse / rabbit IgG bound to Alexa Fluor 488 (1:500, #ab150113 / #ab150077, Abcam). Nuclear staining was performed with DAPI, and fluorescence images were obtained using a Zeiss inverted LSM confocal microscope (Carl Zeiss).

### Cell invasion and migration analysis

Cells (2×10^4^) suspended in medium without FBS were plated on the upper chamber membranes (8 µm pore size, Corning) coated with or without Matrigel (BD Biosciences). The inserts were incubated in a medium supplemented with 10% FBS for 16 h. To evaluate the invasion and migration ability, non-invasive or non-migrative cells were removed. Invasive and migrative cells were fixed with methanol, stained with 0.5% crystal violet, and counted under a light microscope.

### Colony formation assay

Cells were seeded at a density of 1000 per well in 6-well formats with at least three replicates per condition. After 14 days, cells were fixed with 4% paraformaldehyde and stained with 0.5% crystal violet. Cell colonies were imaged and counted for quantification.

### Cell cycle and apoptosis assays

These assays were performed using fluorescence-activated cell sorting (FACS). Cells were treated with the drug for 24 h. For the cell cycle assay, cells were fixed in cold 70% ethyl alcohol for 30 min at 4 °C and then incubated with propidium iodide (PI) for 15 min at room temperature. Apoptosis was assessed using an Annexin V-FITC/PI Apoptosis Detection kit (Yeasen) according to the manufacturer's instruction. Fluorescence was detected using a FACSCanto flow cytometer (Becton Dickinson) and analyzed with FlowJo software.

### GTPases pulldown assay

Rap1A and RhoA pulldown assays were performed by using Rho Activation Assay kit (Cytoskeleton) and Rap1 Activation Assay kit (ThermoFisher), respectively. Briefly, cells were cultured until reaching approximately 90% confluence, then starved in serum-free medium for 24 h and stimulated with 10% FBS for 10 minutes. Cells were lysed in GST-pulldown lysis buffer. For RhoA or Rap1A pulldown, clarified lysates were incubated with Rhotekin or RalGDS RBD Agarose beads for 1 h at 4 °C. Beads were pelleted by centrifugation and washed three times with the washing buffer. Bound GTPases (activated RhoA or Rap1A) were detected by immunoblotting with anti-RhoA or anti-Rap1A antibodies.

### RNA-Seq analysis

RNA-Seq and differentially expressed gene analysis were performed as previously described 28. Raw sequencing reads were mapped to the human genome (hg38) and GENCODE v38 using hisat2. Mapped reads were assigned to genes by featureCounts from Rsubread. Differentially expressed genes were calculated byDESeq2. Functional annotations were done using clusterProfiler. Gene set enrichment analysis (GSEA) was performed using the Broad GSEA application.

### RNA extraction and quantitative real-time PCR

Total RNA was isolated from cells using TRIzol reagent (Invitrogen) according to the manufacturer's instruction. Total RNA from each sample was reverse transcribed to cDNA using the PrimeScript RT kit (Takara). Quantitative real-time PCR (qPCR) was performed on a Roche Light Cycler 480 System (Roche) using the SYBR Premix Ex Taq II RT-PCR Kit (Takara) to determine the transcript levels of the target genes. The primers were listed in Table S3. Results were analyzed using the 2^-(△△Ct)^ method with ACTB expression as an internal control.

### Exosome isolation and analyses

Exosomes were isolated from cell culture medium by differential centrifugation according to previous publications 29, 30. In brief, cells were removed from cell culture supernatant by centrifugation at 300 *g* and 3000 *g* for 10 min to remove any cell contamination. To remove any possible apoptotic bodies and large cell debris, the supernatants were then spun at 10,000 *g* for 30 min. Finally, exosomes were collected by spinning at 100,000 *g* for 70 min. Exosomes were washed in 30 ml PBS and pelleted again by ultracentrifugation (Beckman XPN-100). The concentration and size distribution of exosomes were directly tracked using the Nanosight NS 300 system (NanoSight Technology).

### Enzyme linked immunosorbent assay (ELISA)

The concentration of human ITGA6 was measured using an ELISA kit (ELK Biotechnology) following the manufacturer's instruction. Briefly, peripheral blood samples were centrifuged at 1500 *g* for 20 min, and the supernatant was collected. Diluted standards and samples were added to the pre-coated microplate. Absorbance (OD value) was measured using a microplate reader (Promega), and sample concentrations were calculated by constructing a standard curve.

### Drug sensitivity assays

Cells were seeded into triplicate wells of a 96-well plate at an initial density of 3000 cells per well. After 24 h, cells were treated with different doses of 5-FU, Oxaliplatin, Docetaxel and Y16. Sensitivity was assayed using CellTiter-Glo (Promega), which measures cellular ATP levels as a surrogate for cell number and growth, according to the manufacturer's protocol. Luminescence was measured using a PerkinElmer Envision. Half-maximal inhibitory concentration (IC_50_) values were determined from fitted concentration-response curves obtained from three independent experiments using GraphPad Prism 9 nonlinear regression curve fit.

### Mouse experiments

All animal experiments were conducted under the Institutional Animal Care and Use Committee (IACUC)-approved protocols at Ren Ji Hospital, Shanghai Jiao Tong University School of Medicine in accordance with NIH and institutional guidelines. NOD/SCID female mice aged 6-8 weeks (SLAC, Shanghai, China) were randomly divided into subgroup. For the subcutaneous model, 1×10^6^ MFC cells mixed with 1×10^6^ normal fibroblasts (NFs) or cancer-associated fibroblasts (CAFs) were injected subcutaneously into mice (6 mice for each group). For the lung metastasis model, 1×10^6^ gastric cancer cells were injected into the tail vein of mice (10 mice for each group). For the peritoneal metastasis model, 5×10^6^ cells were injected intraperitoneally into mice (6 mice for each group). For subcutaneous tumors, tumor sizes were determined by measuring the length and width every two days, tumor volumes were calculated according to the following formula: volume (mm^3^) = (length × width^2^)]/2. For in vivo drug treatment, 7 days after injection, tumor-bearing mice were treated randomly with vehicle, Docetaxel (5 mg/kg), Y16 (20 mg/kg), and combination intraperitoneally every day for 21 days. After injection of D-luciferin, bioluminescence imaging of each mouse was performed and analyzed using the IVIS Lumina imaging station (Caliper Life Sciences). Mice were euthanized when came to experimental endpoints and time points, then tissues (tumor, lung, liver, ovary) were removed and analyzed.

### Statistical analysis

Statistical analyses were performed using GraphPad Prism 8.3 software. The results were presented as the mean ± SD. Two-tailed Student's *t*-test was used for comparisons between two groups and one-way ANOVA was used for comparisons between multiple groups. Kaplan-Meier survival analysis was carried out using the log-rank test. *P* < 0.05 was considered statistically significant.

## Results

### Genomic heterogeneity of primary gastric cancer tumors and ovarian metastases

**Figure 1:**
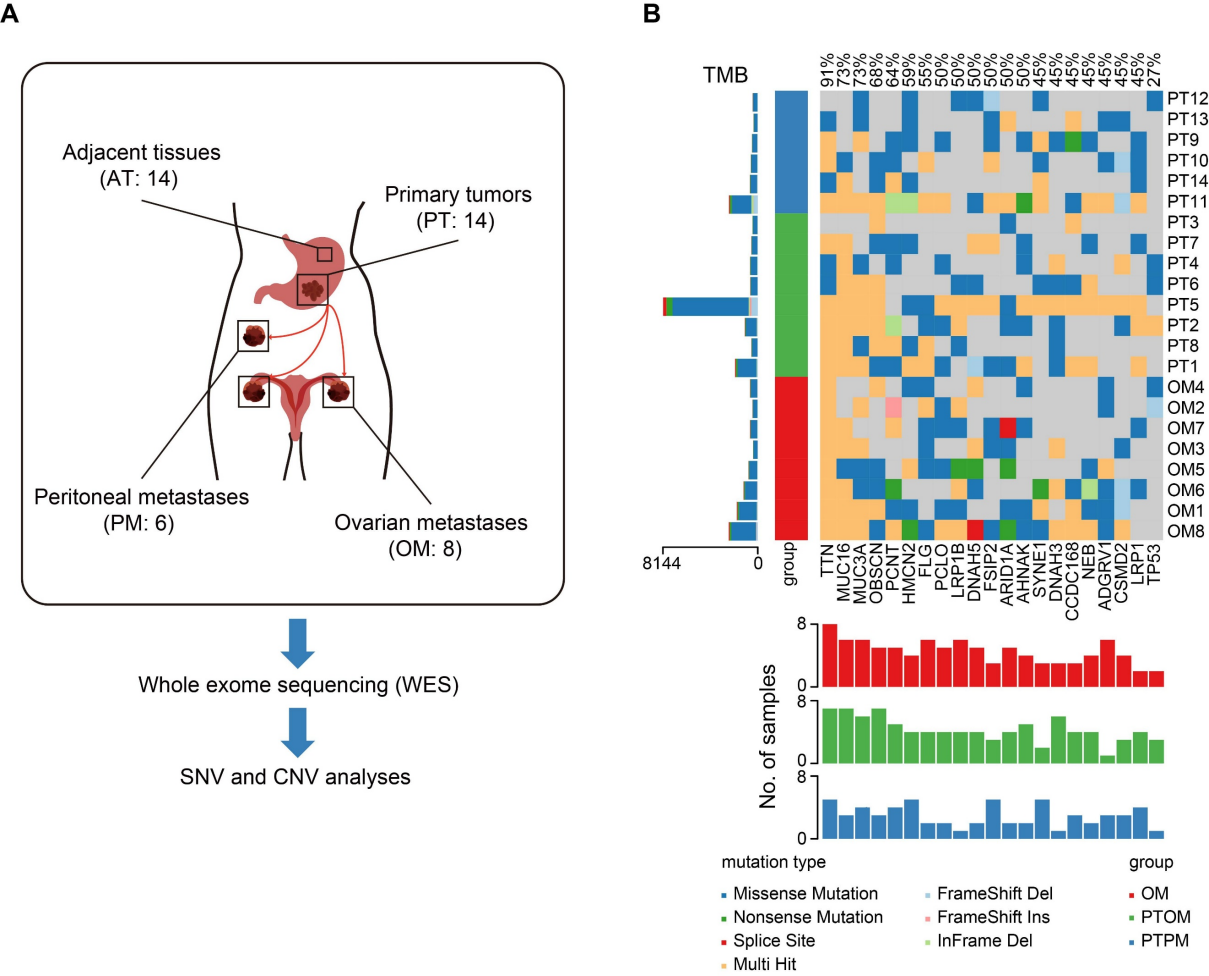
** Genomic heterogeneity of primary gastric cancer tumors and ovarian metastases.** (A) Schematic diagram of WES process in this study. (B) Somatic genomic alterations identified across samples. The main panel shows a matrix of mutations, with distinct colors representing different mutation types. The left histogram illustrates the tumor mutation burden (TMB) for each individual sample. The left track indicated sample types. The bottom histogram summarizes the cumulative number of alterations observed across the samples.

To identify mutations specifically associated with ovarian metastasis, a total of 14 gastric cancer (GC) patients with ovarian metastasis (OM, n = 8) and peritoneal metastasis (PM, n = 6) from Renji hospital were enrolled in the study. All patients underwent gastrectomy and metastasectomy as primary treatment and all specimens were collected prior to chemotherapy. We performed whole-exome sequencing (WES) on 14 GC primary tumors (PT), with matched adjacent tissues (AT) and distant metastases, to validate molecular alterations associated with OM (Figure 1A). The three sample groups were distinguished through principal component analysis (PCA) of the mutational landscape (Figure S1A). Mutation frequencies were higher in primary tumors with ovarian metastasis (PTOM) compared to those with peritoneal metastasis (PTPM) (Figure S1B-C). Additionally, mutation frequencies in PTOM were higher than matched OM (Figure S1D). We identified a total of 3806 single-nucleotide variants (SNVs) using MuTect2, with an average of 1810 per sample. The incidence of top 21 recurrent mutations was shown in Figure 1B, including several frequently mutated genes previously reported in GC with OM, such as *TNN* (12/14), *MUC16* (10/14), *FLG* (6/14), *ARID1A* (6/14) 13. The difference of ratios of mutations across sample groups were not significant (Figure S1E), suggesting that the heterogeneity of mutation accumulation among AT, PT and OM exhibits no significant differences. We further analyzed copy number variations (CNVs) and found that the number of gain (> 4) or loss (< 2) of CNVs showed no significant differences between PTOM and OM (Figure S1F).

### Identification of key mutations related to GC with OM

To identify the driver mutations responsible for OM, we first compared the mutation profiles between PT and OM, and a list of de novo mutations were uniquely found in the OM (Figure S2A). Gene Ontology (GO) enrichment analysis of the mutated genes revealed significant enrichment in pathways related to cell adhesion and mitochondrial calcium transportation (Figure S2B).

**Figure 2:**
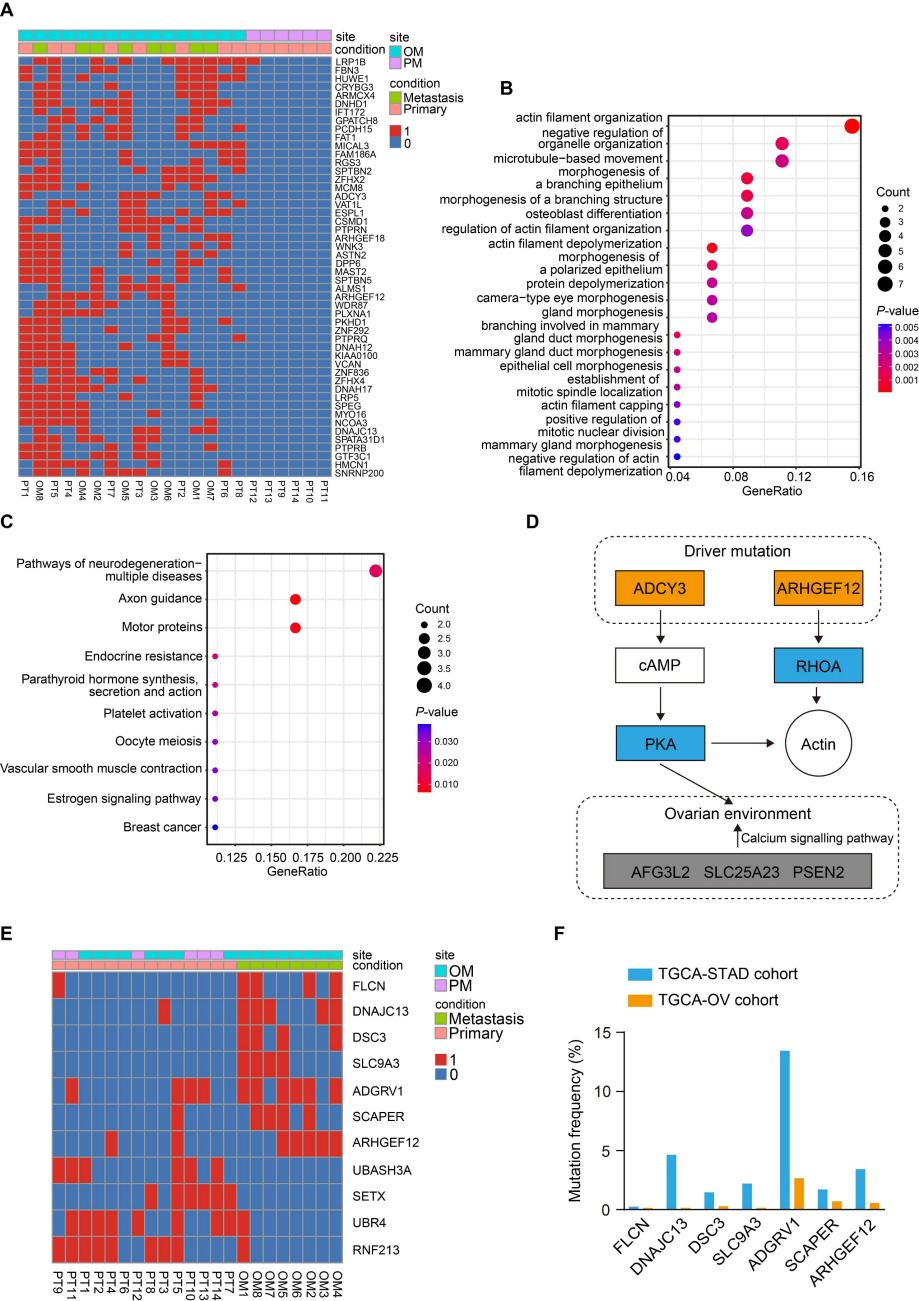
** Identification of key mutations related to gastric cancer (GC) with ovarian metastasis (OM).** (A) Heatmap of specifically driver mutations in GC with OM compared to peritoneal metastasis (PM). 0, non-mutation. 1, mutation. (B) Gene Ontology (GO) enrichment analysis of driver mutations in GC with OM. (C) Kyoto Encyclopedia of Genes and Genomes (KEGG) pathway analysis of driver mutations in GC with OM. (D) Schematic of two driver mutated genes and their mediated genes, thereby regulating actin organization and the ovarian microenvironment through related signaling pathways. Mutated components are indicated by the colored boxes. (E) Heatmap of significantly recurrent mutations from primary tumor (PT) sites and OM sites. (F) Mutation frequencies of genes identified from OM sites in the TCGA-STAD and TCGA-OV cohorts.

We further compared the mutation profiles of PT that resulted in OM with those that led to PM (Figure 2A). GO enrichment analysis of the mutated genes revealed that they were primarily enriched in pathways related to actin filament organization (Figure 2B). Kyoto Encyclopedia of Genes and Genomes (KEGG) enrichment analysis identified significant involvement of the axon guidance and estrogen signaling pathways (Figure 2C), suggesting that the ovarian microenvironment may influence this process. Notably, *ADCY3* and *ARHGEF12* were mainly enriched in these signaling pathways (Figure S2C), indicating that mutations in these genes may be functionally implicated (Figure 2D). ADCY3 encodes the enzyme adenylate cyclase 3, catalyzing the conversion of ATP to cAMP, which serves as a secondary messenger 31. Elevated cAMP activates its effector, protein kinase A (PKA), regulating various biological processes through phosphorylation of substrate proteins 32. ADCY3 has been reported to promote gastric cancer tumorigenesis and is also associated with ovarian development and disease 33-35. ARHGEF12 encodes a Rho guanine nucleotide exchange factor (GEFs) and regulates cytoskeleton dynamics, cell polarization, and migration through activating Rho GTPases 15-17.

Finally, we compared the mutation profiles between PT and OM using the DESeq2 and identified seven mutated genes from the OM sites (*P* <0.05), including *FLCN*,* DNAJC13*,* DSC3*,* SLC9A3*,* ADGRV1*,* SCAPER* and* ARHGEF12* (Figure 2E). We further downloaded The Cancer Genome Atlas Stomach Adenocarcinoma (TCGA-STAD) and The Cancer Genome Atlas Ovarian Cancer (TCGA-OV) datasets and found that mutations in these genes were recurrent in both cohorts (Figure 2F). The mutation rates of *ADGRV1* (Adhesion G Protein-Coupled Receptor V1) were the highest among seven genes in both GC and ovarian cancer, with 13.45% (55 out of 409) and 2.66% (19 out of 705) of samples, respectively (Figure 2F). The mutations rates of* ARHGEF12* were relatively high, with 3.42% (14 out of 409) and 0.56% (4 out of 705) of samples, respectively (Figure 2F). Taken together, these data indicate that the seven mutated genes play a crucial role in OM of GC and may represent potential predictive and therapeutic targets.

### Mutation of *ARHGEF12* is correlated with OM of GC

**Figure 3:**
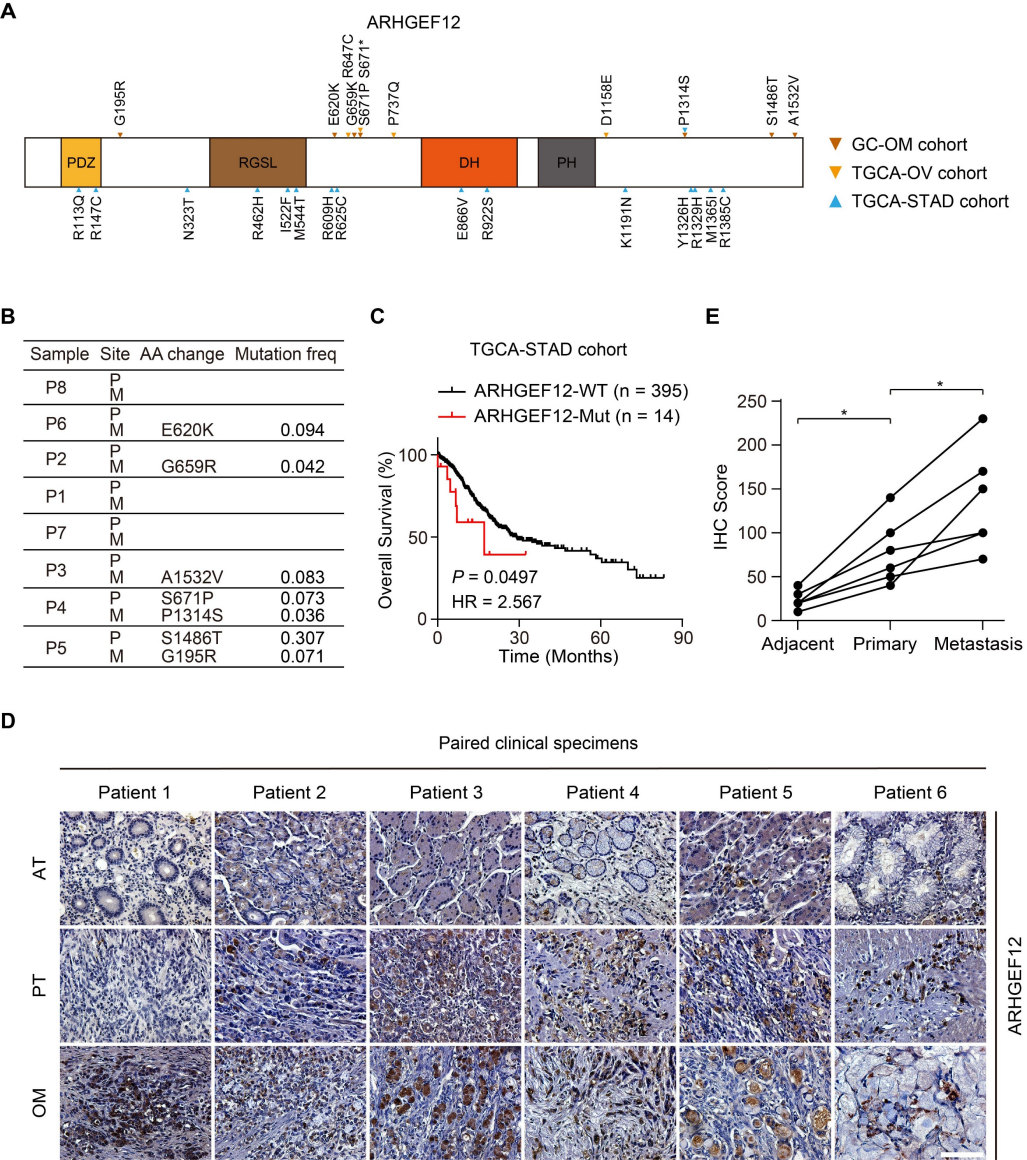
** Mutation of *ARHGEF12* is correlated with OM of GC.** (A) Schematic of protein alterations in ARHGEF12. All alterations listed have been validated by Sanger sequencing. (B) Mutation frequency of *ARHGEF12* in GC with OM. (C) Kaplan-Meier analysis of overall survival (OS) in GC patients with mutation (MUT) or wildtype (WT) *ARHGEF12* from the TCGA-STAD cohort. (D) Representative images of immunohistochemistry (IHC) staining showed ARHGEF12 expression in six matched ATs, PTs and OMs. Scale bars, 100 µm. (E) Quantification of ARHGEF12 expression in (D). Data were presented as mean ± SEM. *P < 0.05, by paired two-tailed *t*-test or log-rank test.

Given the relatively high recurrence rate of *ARHGEF12* mutations in GC and ovarian cancer, and its enrichment in signaling pathways related to GC with OM, we further investigated the functions of *ARHGEF12* mutations in GC. Initially, we validated seven *ARHGEF12* missense mutations in our cohort, with two originating from the PT and five from the OM (Figure 3A-B). Subsequently, we analyzed* ARHGEF12* mutation frequency across multiple cancer types in the Cosmic dataset and found that gastric cancer exhibited the second highest *ARHGEF12* mutation frequency (6.16%, 100 out of 1623) among various cancer types (Figure S2D). Finally, we downloaded the TCGA-STAD dataset and revealed that recurrent mutations of *ARHGEF12* were associated with poor prognosis (Figure 3C).

Next, we performed immunohistochemistry (IHC) analysis on six PT, with matched AT and OM, to evaluate the protein expression levels of ARHGEF12. The protein levels of ARHGEF12 were higher in PT compared to the matched AT, and the highest protein levels were observed in the matched OM (Figure 3D-E). Additionally, ARHGEF12 expression levels were significantly higher in PT compared to the AT in the TCGA-STAD cohort (Figure S2E). ARHGEF12 expression was significantly elevated in stage IV patients compared to stages I-III (Figure S2F), suggesting that elevated ARHGEF12 levels are positively correlated with distant metastasis. Importantly, high ARHGEF12 expression was significantly associated with poor prognosis in GC, as indicated by the KM Plotter database (Figure S2G). These findings suggest that *ARHGEF12* mutation may contribute to OM of GC and ARHGEF12 could act as a prognostic factor.

### *ARHGEF12* depletion decreases cell migration capacity in GC

**Figure 4:**
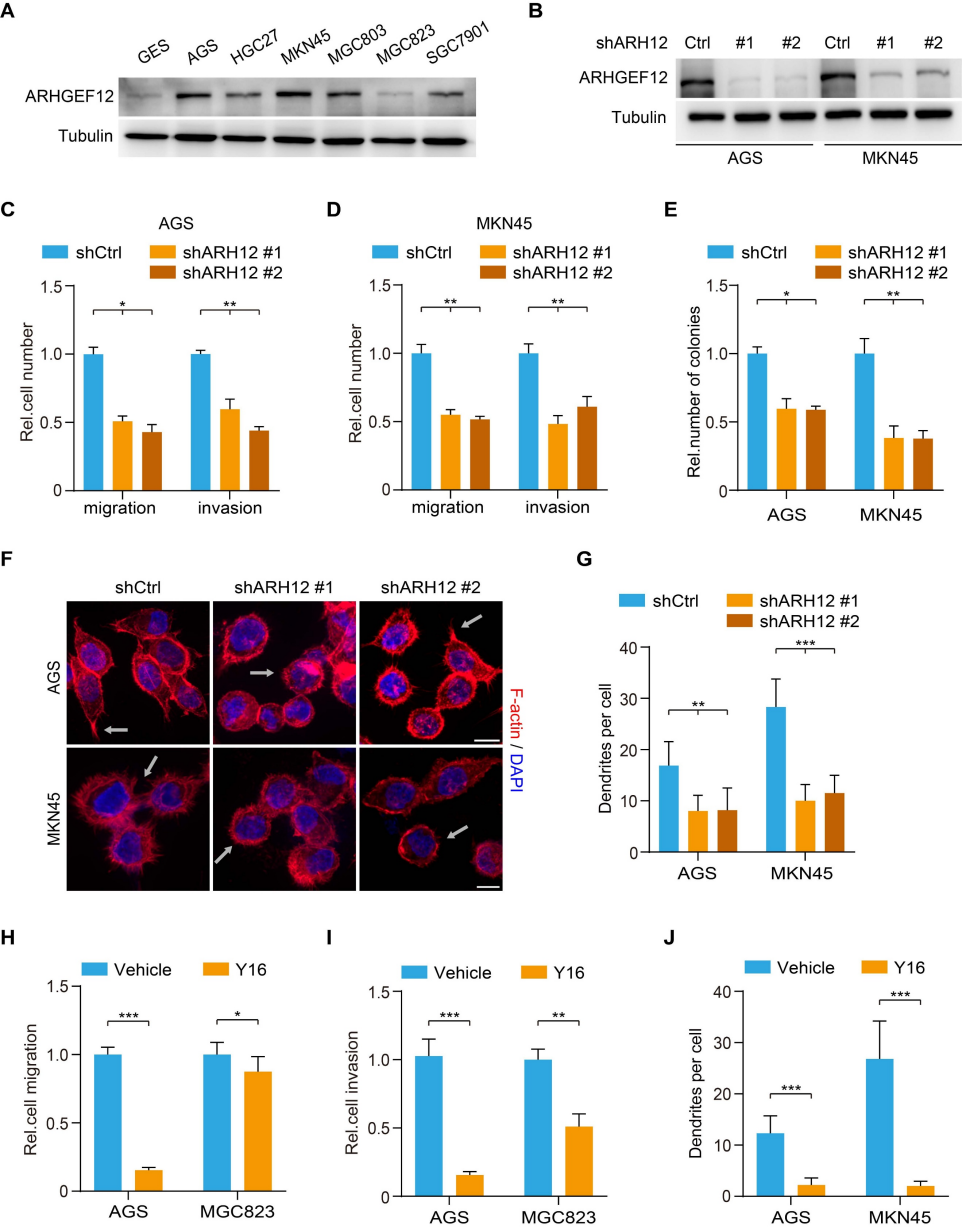
***ARHGEF12* depletion decreases cell migration capacity in GC.** (A) Western blotting (WB) of ARHGEF12 expression in gastric mucosal epithelial cell line GES and six gastric cancer cell lines. (B) WB of *ARHGEF12* knockdown (KD) in AGS and MKN45 cells. A control shRNA (shCtrl) and two different ARHGEF12 shRNAs (shARH12 #1 and shARH12 #2) were used. (C-E) Effects of *ARHGEF12* KD on AGS and MKN45 cells migration (C), invasion (D) and colony formation (E). (F) Representative immunofluorescence (IF) images of AGS and MKN45 cells with or without *ARHGEF12* KD. Red: F-actin, Blue: DAPI. Scale bar, 10 µm. (G) Quantification of the number of filopodia. Data shown are from 30 cells counted per condition. (H-I) Cell migration (H) and cell invasion (I) analysis of AGS cells (high ARHGEF12 expression) or MGC823 cells (low ARHGEF12 expression). Cells were pre-treated with or without 50 µM Y16 for 48 h and then subjected to cell migration and cell invasion assays. (J) Quantification of the number of filopodia. Data shown are from 30 cells counted per condition. Data are representative of three independent experiments with similar results. Data were presented as mean ± SEM. *P < 0.05, **P < 0.01, ***P < 0.001, by two-tailed Student's *t*-test or one-way ANOVA analysis.

To further investigate the biological function of ARHGEF12 in GC, we initially assessed ARHGEF12 expression in a gastric epithelial cell line GES and various GC cell lines. Compared to GES cells, ARHGEF12 protein was highly expressed in most GC cell lines (Figure 4A). Subsequently, we knockdown *ARHGEF12* with two different shRNAs, shARH12#1 and shARH12#2 in AGS and MKN45 cells (Figure 4B). *ARHGEF12* knockdown (KD) markedly reduced cell migration, invasion, and colony formation (Figure 4C-E), but had no significant effect on cell proliferation (Figure S3A-B). Moreover, *ARHGEF12* KD disrupted actin cytoskeleton organization and reduced the number of filopodia, thereby impairing cell migration (Figure 4F-G).

We further treated GC cells with a small molecule inhibitor Y16 of ARHGEF12 36, 37. Y16 (50 µM) treatment significantly suppressed cell proliferation, migration, and invasion in AGS cells with high ARHGEF12 expression, while exhibiting a modest inhibitory effect on MGC823 cells with low ARHGEF12 expression (Figure 4H-I, Figure S3C-D). Treated AGS and MKN45 cells with Y16 also disrupted the actin cytoskeleton organization and reduced the number of filopodia (Figure 4J). The inhibitory effect of Y16 treatment was weaker on *ARHGEF12* KD cells than on control cells, suggesting that Y16 suppresses cell proliferation primarily by targeting ARHGEF12 (Figure S3E-F). Additionally, we assessed and revealed that a low concentration of Y16 (10 µM) had no significant effect on cell proliferation (Figure S3G), whereas it suppressed cell migration and invasion in AGC cells (Figure S3H). Moreover, treatment with 50 µM Y16, but not 10 µM Y16, arrested the cell cycle at G2/M phase and induced apoptosis, thereby suppressing cell proliferation (Figure S3I-K). These results demonstrate that *ARHGEF12* is critical for cell migration and invasion in GC.

### E620K mutation of *ARHGEF12* promotes OM in GC

**Figure 5:**
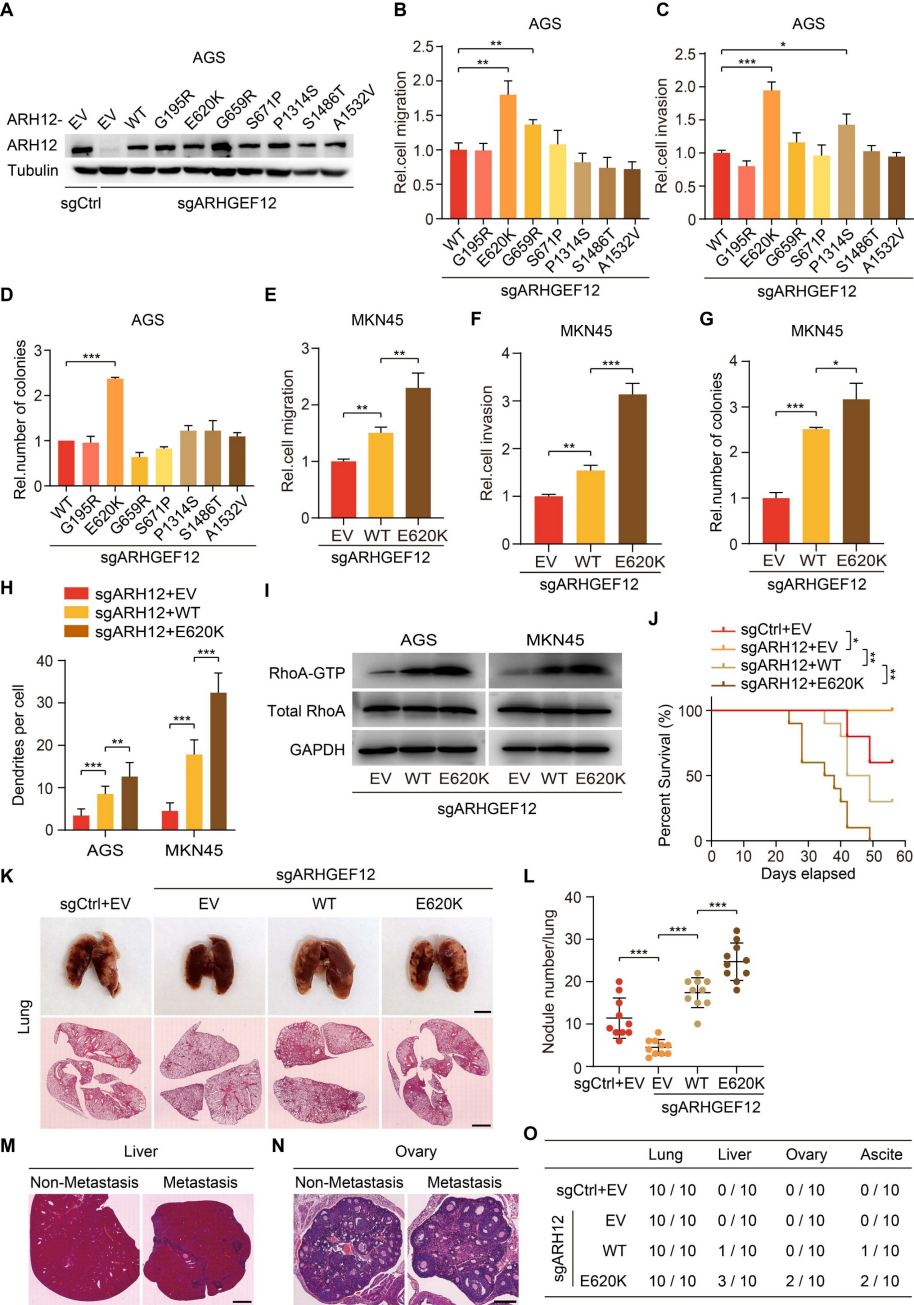
** E620K mutation of *ARHGEF12* promotes OM in GC.** (A) WB of *ARHGEF12* knockout (KO) and *ARHGEF12* mutants. A control sgRNA (sgCtrl) and one ARHGEF12 sgRNA (sgARHGEF12) were used. Empty vector (EV), wild-type (WT) *ARHGEF12* or* ARHGEF12* mutants were re-expressed in *ARHGEF12* KO AGS cells. ARH12, ARHGEF12. (B-D) Effects of *ARHGEF12* mutations on cell migration (B), invasion (C) and colony formation (D). (E-G) Effects of *ARHGEF12* E620K mutation on cell migration (E), invasion (F) and colony formation (G) in MKN45 cells. (H) Quantification of filopodia numbers affected by *ARHGEF12* E620K mutation in AGS and MKN45 cells. Data shown are from 30 cells counted per condition. (I) Immunoblotting for the active RHOA by Rhotekin pulldown assay. Cells were serum starved for 24 h and stimulated with 10% FBS for 10 min. Total cell lysates were subjected to GST-Rhotekin Rho-binding domain (RBD) precipitation and then the activities of RhoA were examined. (J) Kaplan-Meier survival of animals with indicated AGS tumors (n = 10). (K) Representative bright-field (upper panel) and hematoxylin and eosin (H&E) staining (lower panel) images of the animal lungs. Scale bars, upper, 300 µm, lower, 100 µm. (L) The numbers of nodules on the lung surfaces in (K) quantified by necropsy. (M) Representative H&E staining images of the animal livers with non-metastasis and metastasis tumors. Scale bars, 100 µm. (N) Representative H&E staining images of the animal ovaries with non-metastasis and metastasis tumors. Scale bars, 20 µm. (O) Detailed summary of the tumorigenicity and peritoneal metastasis of animals. Data are representative of three independent experiments with similar results. Data were presented as mean ± SEM. *P < 0.05, **P < 0.01, ***P < 0.001, by two-tailed Student's *t*-test or log-rank test.

To explore the functional effects of *ARHGEF12* mutation discovered from our analysis, we established *ARHGEF12* knockout (KO) AGS cells using two sgRNAs and then transduced wild-type (WT) *ARHGEF12* or seven mutants G195R, E620K, G659R, S671P, P1314S, S1486T and A1532V into AGS/sgARHGEF12 cells, respectively (Figure 5A). Comparison with WT *ARHGEF12*, only the E620K mutation significantly increased cell migration, invasion, and colony formation in AGS cells (Figure 5B-D), while having no significant effect on cell proliferation (Figure S4A). Consistent results were observed in MKN45 cells (Figure 5E-G, Figure S4B). Moreover, re-expression of* ARHGEF12* E620K mutant significantly increased cell filopodia formation reduced by *ARHGEF12* KO in AGS and MKN45 cells (Figure 5H, Figure S4C). We next performed GTPase pulldown assay to detect active RhoA (RhoA-GTP) levels. Re-expression of* ARHGEF12* E620K mutant significantly elevated RhoA-GTP levels compared to WT *ARHGEF12*, with no corresponding change in total RhoA protein levels (Figure 5I). These data suggest that *ARHGEF12* E620K mutation represents a gain-of-function mutation, enhancing the invasive potential of gastric cancer cells through hyperactivation of the canonical GTPase RhoA.

To further investigate the functions of *ARHGEF12* E620K mutation in vivo, we transplanted AGS cells into nude mice via tail vein injection. Animals were sacrificed 4-8 weeks post injection. *ARHGEF12* KO extended animal survival compared to controls (Figure 5J), and re-expression of *ARHGEF12* WT reversed the prolonged animal survival observed in *ARHGEF12* KO group (Figure 5J). Compared to *ARHGEF12* WT, re-expression of *ARHGEF12* E620K mutant significantly reduced animal survival (Figure 5J). We collected lung, liver, ovary, and ascites for analysis.* ARHGEF12* KO decreased metastatic lung nodule formation compared to the controls (Figure 5K-L) and re-expression of *ARHGEF12* WT reversed *ARHGEF12* KO-reduced metastatic lung nodule formation (Figure 5K-L). Compared to *ARHGEF12* WT, re-expression of *ARHGEF12* E620K mutant significantly promoted metastatic lung nodule formation (Figure 5K-L). Strikingly, ARHGEF12 E620K mutant exhibited more aggressive, with three of ten mice developing liver metastasis, and two of these three mice developing OM and ascites (Figure 5M-O). These data demonstrate that *ARHGEF12* E620K mutation promotes OM in GC.

### *ARHGEF12* E620K mutation activates Rap1 signaling pathway and upregulates integrin gene expression

**Figure 6:**
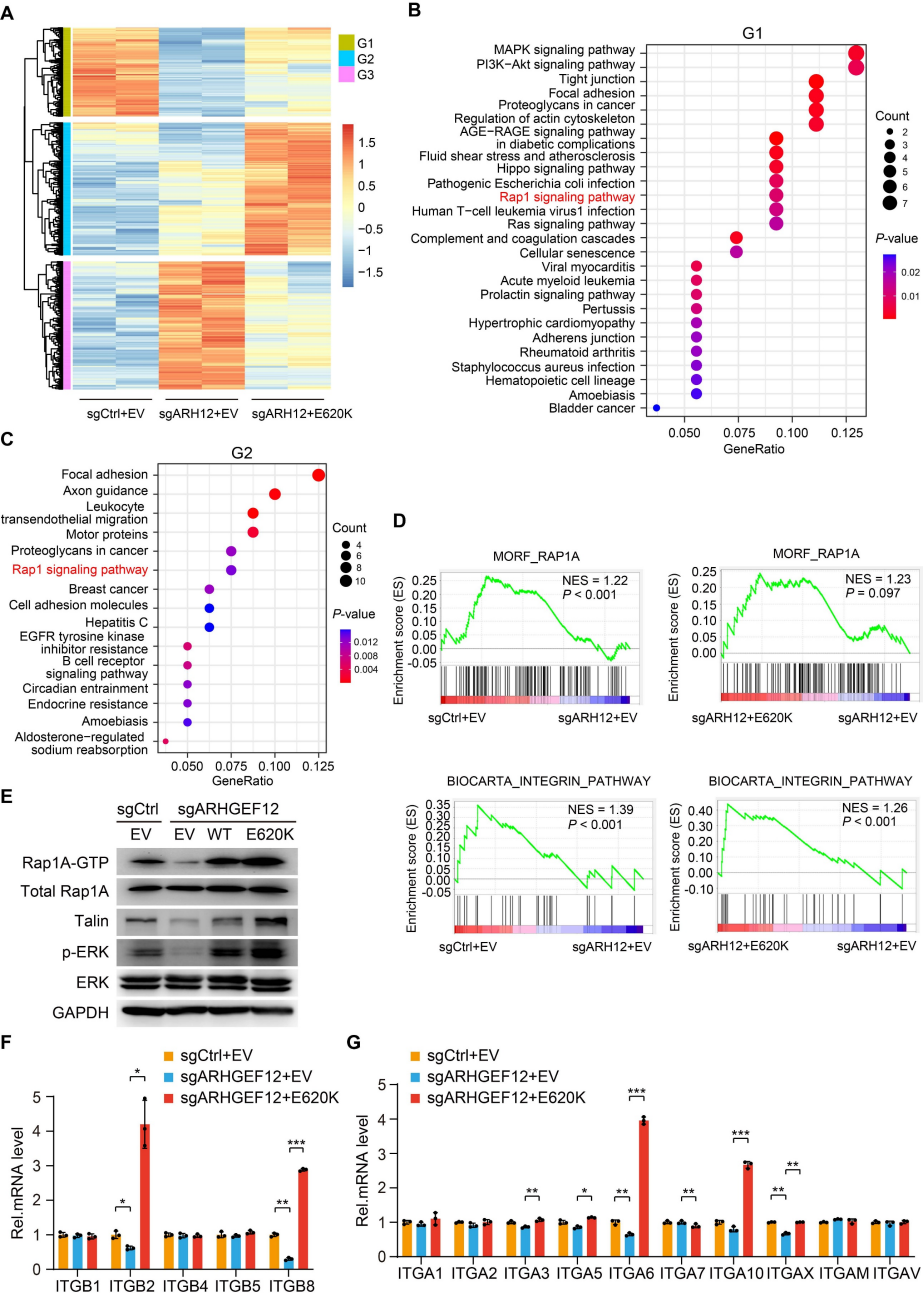
***ARHGEF12* E620K mutation activates Rap1 signaling pathway and upregulates integrin gene expression.** (A) Heatmap of mRNA-Seq analysis of differentially expressed genes regulated by *ARHGEF12* mutant E620K (fold change >2, *P* <0.05). (B-C) GO enrichment analysis of downregulated genes (G1) by *ARHGEF12* KO (B) or upregulated genes (G2) by the E620K mutant (C). (D) Gene set enrichment analysis (GSEA) showed enrichment of Rap1 signaling pathway and integrin-related gene signature in E620K mutated cells. NES, normal enrichment score. (E) WB of effects of *ARHGEF12* E620K mutation on Rap1 signaling activation (Rap1A-GTP, Talin, and p-ERK) in AGS cells. (F-G) qRT-PCR analysis of relative mRNA levels of integrin α (F) and β (G) family members regulated by the E620K mutant in AGS cells. Data are representative of three independent experiments with similar results. Data were presented as mean ± SEM. *P < 0.05, **P < 0.01, ***P < 0.001, by two-tailed Student's *t*-test.

To explore the mechanism by which *ARHGEF12* E620K mutation promotes OM, we performed RNA-Seq analysis on AGS cells transduced with sgCtrl, sgARHGEF12, or re-expressed *ARHGEF12* E620K mutant. We identified a total of 188 genes were significantly downregulated following *ARHGEF12* KO and a total of 281 genes were significantly upregulated by re-expression of the E620K mutant (fold change >2, *P* <0.05) (Figure 6A). KEGG pathway analysis revealed that MAPK signaling, PI3K-Akt signaling, Hippo signaling, Rap1 signaling, Ras signaling, tight junction, and focal adhesion were enriched in the genes downregulated by* ARHGEF12* KO (Figure 6B). Re-expression of the E620K mutant not only upregulated the pathways downregulated by *ARHGEF12* KO, but further upregulated focal adhesion, axon guidance, and Rap1 signaling compared to the sgCtrl (Figure 6C). We further employed gene set enrichment analysis (GSEA) and found that Rap1A pathway and integrin pathway were enriched by *ARHGEF12* KO-downregulated genes and the E620K mutant-upregulated genes (Figure 6D). These data indicate that *ARHGEF12* E620K mutation activates Rap1A pathway and integrin pathway.

Since Rap1 is involved in integrin-mediated cell adhesion, cytoskeleton rearrangement, and cell polarity 38-40 and *ARHGEF12* selectively activates Rap1A in human dermal microvascular endothelial cells 41, we hypothesized that *ARHGEF12* E620K mutation activates Rap1A pathway to upregulate integrin gene expression. To verify this hypothesis, we first examined the protein levels of Rap1A and downstream effectors Talin and ERK. As shown in Figure 6E,* ARHGEF12* KO downregulated active Rap1A (Rap1A-GTP) levels, Talin protein expression and ERK phosphorylation (p-ERK), while re-expression of the E620K mutant significantly restored Rap1A-GTP levels, Talin expression and p-ERK reduced by *ARHGEF12* KO. We then performed qRT-PCR analysis and confirmed that *ARHGEF12* KO decreased mRNA levels of *ITGB2*, *ITGB8*, *ITGA6* and *ITGAX*, while re-expression of the E620K mutant significantly increased the expression of these integrin genes (Figure 6F-G). Notably, *ITGB2* and *ITGA6* were identified as differentially expressed genes following *ARHGEF12* KO or re-expression of the E620K mutant (Figure 6A). *ITGB2* exhibited the most significant change among all integrin genes (Figure S5A-B). These data demonstrate that *ARHGEF12* E620K mutation activates Rap1 signaling pathway and upregulates integrin gene expression.

### Tumor-derived ITGA6-high exosomes are predominantly uptaken by ovarian fibroblasts

**Figure 7:**
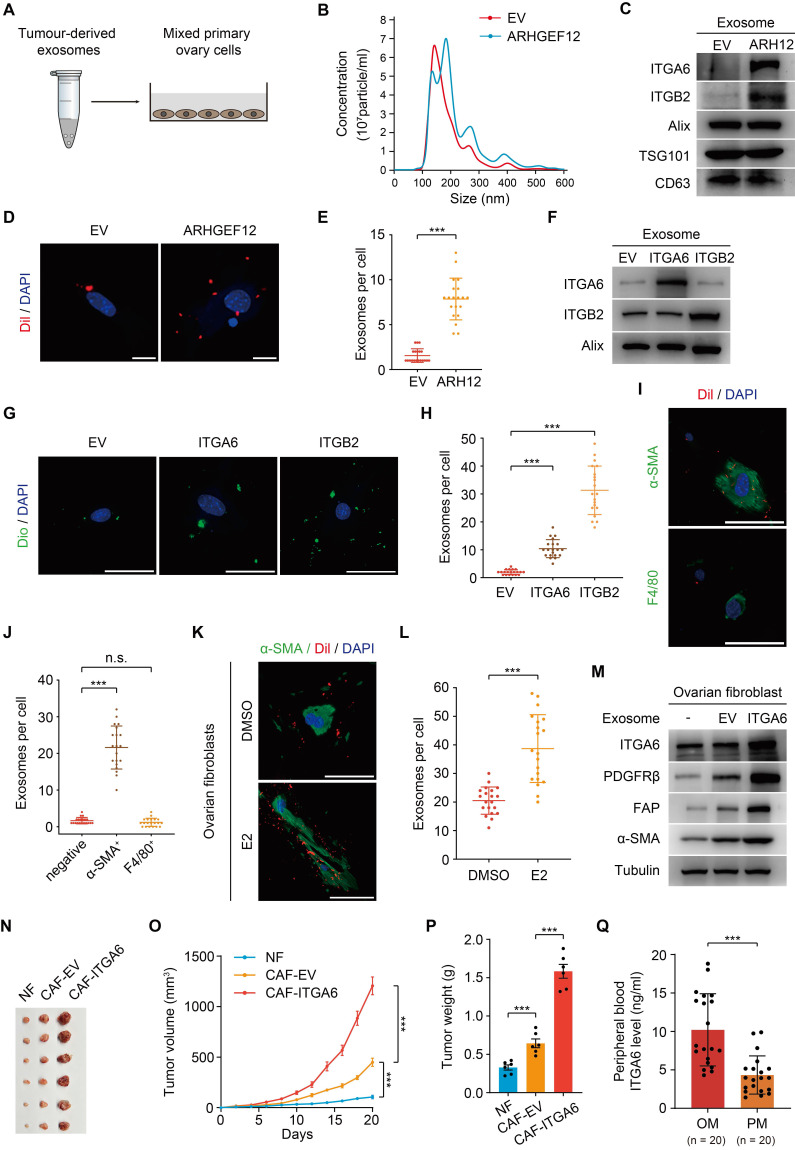
** Tumor-derived ITGA6-high exosomes are predominantly uptaken by ovarian fibroblasts.** (A) Schematic representation of the experimental design. Exosomes derived from mouse forestomach carcinoma (MFC) cells (50 µg) were co-cultured with 2×10^5^ primary ovary cells. (B) Size distribution of tumor-derived exosomes determined by nanoparticle tracking analysis. (C) WB of ITGA6, ITGB2 and exosome markers expression in tumor-derived exosomes. (D) IF staining of primary ovary cells co-cultured with exosomes from MFC cells with EV or ARHGEF12. Scale bars, 50 µm. (E) Quantification of ovary cells uptake of tumor-derived exosomes in (D). Data shown are from 20 cells counted per condition. (F) WB of ITGA6 and ITGB2 expression in exosomes from MFC cells with EV,* ITGA6* or *ITGB2*. (G) IF staining of primary ovary cells co-cultured with ITGA6/ITGB2-high exosomes. Scale bars, 50 µm. (H) Quantification of ovary cells uptake of tumor-derived exosomes in (G). Data shown are from 20 cells counted per condition. (I) IF staining of primary ovary cells co-cultured with exosomes from MFC cells. Fibroblasts were stained with α-SMA and macrophages were stained with F4/80. Scale bars, 50 µm. (J) Quantification of different types of ovary cells that uptake of tumor-derived exosomes in (I). Data represent the counts of 20 positively stained cells per condition. (K) IF staining of mouse ovarian fibroblasts stimulated with estradiol (E2) and co-cultured with exosomes from MFC cells. Fibroblasts were stained with α-SMA. Scale bars, 50 µm. (L) Quantification of mouse ovarian fibroblasts uptake of tumor-derived exosomes in (K). Data shown are from 20 cells counted per condition. (M) WB of the expression of ITGA6 and cancer-associated fibroblasts (CAF) markers α-SMA, FAP and PDGFRβ in ovarian fibroblasts co-cultured with tumor-derived exosomes. (N) Representative image of tumor masses. MFC cells mixed with ovarian normal fibroblasts (NF) or exosome-educated fibroblasts were implanted into animals (n = 6). (O) Tumor growth curves of xenografts in (N). (P) Quantification of tumor weights in (N). (Q) Concentration of ITGA6 in peripheral blood of GC patients with OM or PM (n = 20). Data are representative of three independent experiments with similar results. Data were presented as mean ± SEM. n.s., no significant, ***P < 0.001, by two-tailed Student's *t*-test.

Exosomes are small membrane vesicles (30-100 nm) secreted by tumor cells and have recently been recognized as “primers” of the metastatic niche 42-44. In gastric cancer, EGFR-containing exosomes derived from cancer cells have been shown to create a liver-like microenvironment, thereby promoting liver-specific metastasis 30. However, the contribution of exosomes in predisposing OM remains unexplored. We first isolated exosomes from mouse forestomach carcinoma (MFC) cells transduced with empty vector (EV) or *ARHGEF12*, respectively (Figure 7A). Nanoparticle tracking analysis (NTA) revealed no significant differences in exosome size distribution (Figure 7B). These exosomes expressed typical markers (Alix, TSG101 and CD63). Notably, these exosomes derived from *ARHGEF12*-transduced MFC cells exhibited high ITGA6 and ITGB2 protein levels (Figure 7C). We then co-cultured the exosomes with primary mouse ovarian cells and observed that ovarian cells uptook more exosomes derived from ARHGEF12-overexpressed MFC cells compared to those derived from the control cells (Figure 7D-E).

We further investigated which biomolecules within the exosomes might facilitate their uptaken by ovarian cells. Since integrins play a crucial role in mediating exosome functions, particularly in guiding exosomes to distant organs to form a pre-metastatic niche and further support organ-specific metastasis 45, 46, and our RNA-Seq results revealed that ARHGEF12 upregulates integrins expression, we hypothesized that integrins mediate tumor-derived exosomes. To determine this, we transduced MFC cells with *ITGA6* or *ITGB2*, two integrins significantly upregulated by the E620K mutant (Figure 6F-G). We then isolated exosomes from these cells and confirmed high expression levels of ITGA6 or ITGB2 protein (Figure 7F). When exosomes enriched with ITGA6 or ITGB2 protein were co-cultured with mouse ovarian cells, we observed that the ovarian cells uptook more tumor-derived ITGA6*-* or ITGB2**-**high exosomes compared to those derived from control cells (Figure 7G-H). These data indicate that tumor-derived ITGA6*-* or ITGB2**-**high exosomes are predominantly uptaken by ovarian cells.

Next, we aimed to investigate which type of ovarian cell primarily uptake tumor-derived exosomes. Pervious accumulated evidence indicated that F4/80^+^ macrophage cells and α-SMA^+^ fibroblasts are always considered as recipient cells 29, 30, 47. We performed immunofluorescence (IF) assay and found that α-SMA^+^ fibroblasts, but not F4/80^+^ macrophage cells, predominantly co-localized with tumor-derived exosomes in the ovarian microenvironment (Figure 7I-J). Meanwhile, a recent study reported that ovarian fibroblasts with positive estrogen receptor (ER^+^) promoted OM in GC 14. We isolated mouse ovarian fibroblasts and stimulated them with estradiol (E2), finding that estrogen stimulation enhanced the uptake of tumor-derived exosomes by ovarian fibroblast (Figure 7K-L). Furthermore, considering the frequent occurrence of liver metastasis in animal experiments, we compared exosome uptake efficiency between liver and ovarian fibroblasts. The efficiency of exosome uptaken by liver fibroblasts showed no significant difference compared with ovarian fibroblasts. However, estrogen stimulation failed to enhance exosome uptake ability in liver fibroblasts (Figure S6A-B). Collectively, these findings indicate that tumor cell gene mutations, together with the ovarian microenvironment drive ovarian-specific metastasis.

Previous studies have reported that normal fibroblasts (NFs) internalize exosomes and transform into cancer-associated fibroblasts (CAFs), acquiring a pro-tumorigenic phenotype 48, 49. Consequently, we isolated mouse ovarian NFs and co-cultured with exosomes from MFC cells transduced with *ITGA6* or EV. Fibroblasts educated by exosomes from* ITGA6*-transduced MFC cells exhibited elevated ITGA6 expression (Figure 7M). Meanwhile, exosome-educated fibroblasts showed increased expression levels of α-SMA, FAP, and PDGFRβ, indicating their transformation to CAFs (Figure 7M). Additionally, CAFs with elevated integrins promote tumor cell proliferation, thereby initiating the formation of pre-metastatic niche 50, 51. To examine the function of CAFs in OM, MFC cells were mixed with ovarian NFs or CAFs and were injected subcutaneously into mice. We found that CAFs with high ITGA6 expression significantly increased the tumor growth compared to CAFs with EV or NFs (Figure 7N-P).

Consistently, CAFs educated by exosomes from* ARHGEF12*-transduced MFC cells significantly promoted tumor growth compared with those educated by exosomes from EV-transduced MFC cells (Figure S6C-F). Additionally, we utilized a published single-cell RNA sequencing data to validate our findings 52. This dataset represents two GC patients with OM, containing six matched samples: AT, PT and OM. We examined the expressions of *ITGA6* and *ITGB2* across these samples and found that *ITGA6*, but not *ITGB2*, was highly expressed in fibroblasts in OM compared to AT and PT (Figure S7A-C), supporting that tumor-derived *ITGA6***-**high exosomes are predominantly uptaken by fibroblasts in ovarian tissues. Taken together, these data demonstrate that tumor-derived ITGA6**-**high exosomes are predominantly uptaken by ovarian fibroblasts. Furthermore, ITGA6*-*high exosome-educated fibroblasts in turn exhibited CAFs phenotypes and promoted tumor cell proliferation, thereby initiating the early stage of pre-metastatic niche formation.

Finally, we investigated whether ITGA6 could serve as an early predictive marker. Peripheral blood samples were collected from 20 GC patients with OM and 20 patients with PM. We measured ITGA6 levels through enzyme linked immunosorbent assay (ELISA) experiments and found significantly higher concentrations in OM patients compared to PM patients (Figure 7Q). This result suggests that elevated ITGA6 protein levels in the peripheral blood serum of GC patients are associated with OM, highlighting its potential as a predictive marker.

### Targeting ARHGEF12 with Y16 synergizes with docetaxel to suppress OM

**Figure 8:**
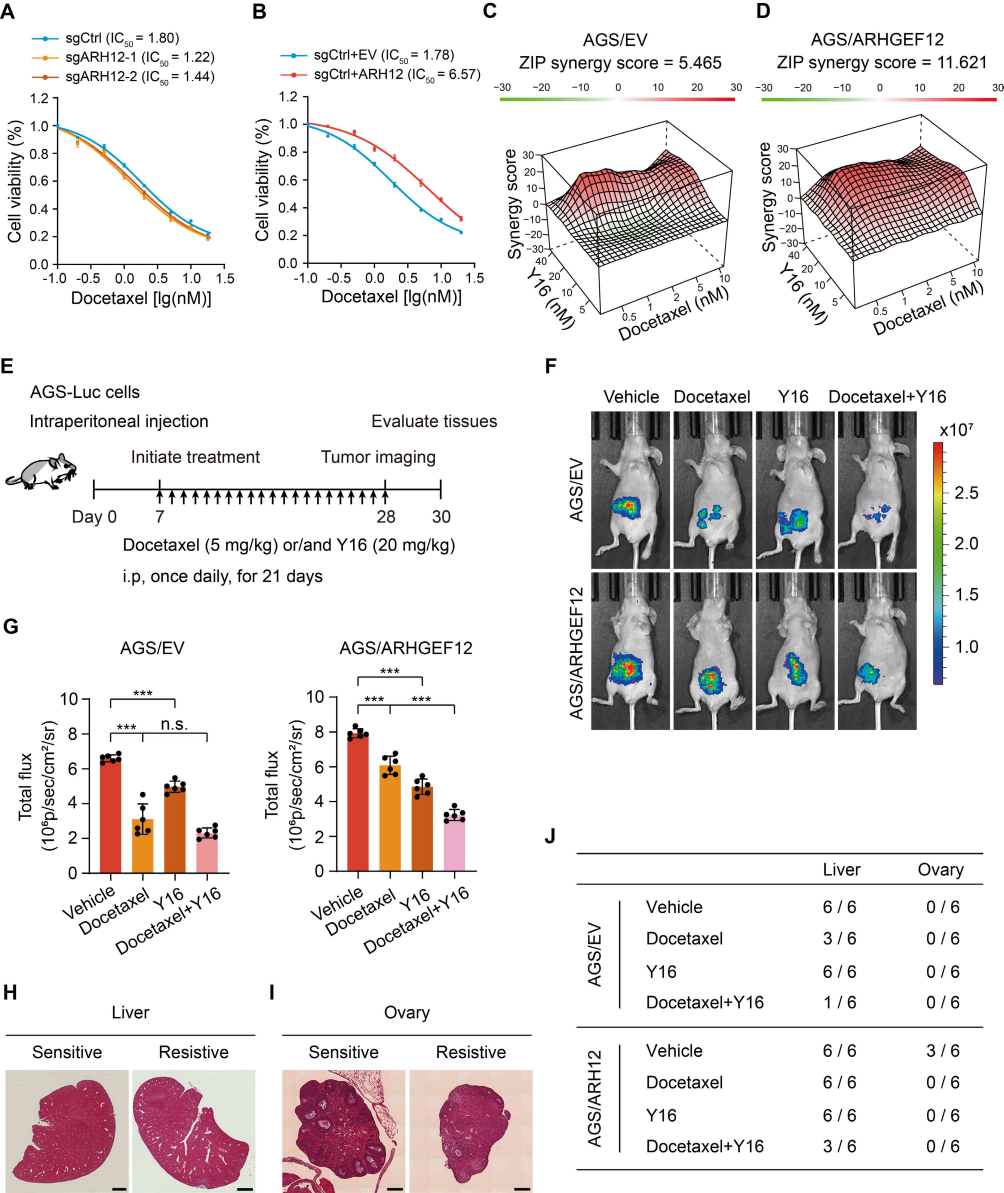
**Targeting ARHGEF12 with Y16 synergizes with docetaxel to suppress ovarian metastasis.** (A) Cell viability analysis. AGS cells with sgARHGEF12 or sgCtrl were treated with docetaxel for 48 h. (B) Cell viability analysis. AGS cells with or without ectopic expression of *ARHGEF12* (AGS/ARHGEF12) were treated with docetaxel for 48 h. (C-D) Synergistic effect analysis. AGS/EV (C) or AGS/ARHGEF12 (D) cells were treated with docetaxel (0.5 to 10 nM) or Y16 (5 to 40 µM), either alone or in combination for 48 h. Viability in the treatment groups was normalized to DMSO control. Analysis of the synergistic effect in docetaxel and Y16 combination was performed by SynergyFinder using zero interaction potency (ZIP) model. The box indicates the synergistic area. (E) Treatment schedules for the administration of docetaxel (5 mg/kg, intraperitoneal injection once daily), or/and Y16 (20 mg/kg, intraperitoneal injection once daily) to animals grafted with AGS/EV or AGS/ARHGEF12 cells (n = 6). Control animals received a placebo (vehicle). (F) Representative bioluminescence images in (E) on day 28. (G) Quantification of bioluminescence activity in panel (F). (H-I) Representative hematoxylin and eosin (H&E) staining images of the animal livers (H) and ovaries (I) with sensitive and resistant treatment. Scale bar, 100 µm and 20 µm. (J) Detailed summary of the peritoneal metastasis of animals after treatment. Data are representative of three independent experiments with similar results. Data were presented as mean ± SEM. n.s., no significant, ***P < 0.001, by two-tailed Student's *t*-test.

Chemotherapy remains the standard treatment strategy for GC patients with OM. To investigate the role of *ARHGEF12* in regulating the growth and chemoresistance of GC cells, we initially treated the cells with 5-FU, oxaliplatin, and docetaxel-three commonly used chemotherapeutic agents for GC treatment. *ARHGEF12* KO significantly increased cell sensitivity to docetaxel but not 5-FU and oxaliplatin (Figure 8A, Figure S8A-B). Consistently, *ARHGEF12* overexpression enhanced the chemoresistance of docetaxel but not 5-FU and oxaliplatin (Figure 8B, Figure S8C-D). These data suggested that high ARHGEF12 expression may contribute to docetaxel chemoresistance in GC.

We then investigated whether targeting ARHGEF12 with Y16 in combination with docetaxel could effectively reduce GC cell proliferation. Using SynergyFinder 53, we detected an additive response (ZIP score = 5.465) between docetaxel and Y16 (Figure 8C, Figure S8E). Additionally, a synergistic response (ZIP score = 11.621) was observed between docetaxel and Y16 in ARHGEF12 overexpression AGS cells (Figure 8D, Figure S8F). These data indicate that the combination of Y16 and docetaxel treatment could suppress chemoresistance in GC due to high ARHGEF12 expression.

To further determine the functions of Y16 inhibitor in docetaxel resistance, we generated a peritoneal metastasis model in immunocompromised mice by intraperitoneally injecting 5×10^6^ AGS-Luc cells. One week post injection, mice were treated with either docetaxel, Y16 alone, or a combination of both for three weeks (Figure 8E). Bioluminescence imaging revealed that the combination treatment significantly reduced peritoneal metastasis compared to the control and single treatment groups, indicating that Y16 synergized with docetaxel to suppressed GC metastasis in vivo (Figure 8F-G). Consistently, combination treatment decreased metastatic nodule formation in liver and ovary, which was attributed to ARHGEF12 overexpression (Figure 8H-J, Figure S8G-H). Taken together, these data suggest that targeting ARHGEF12 with Y16, synergizing with docetaxel in GC with high ARHGEF12 expression, presents a potential novel therapeutic strategy for treating OM.

## Discussion

**Figure 9:**
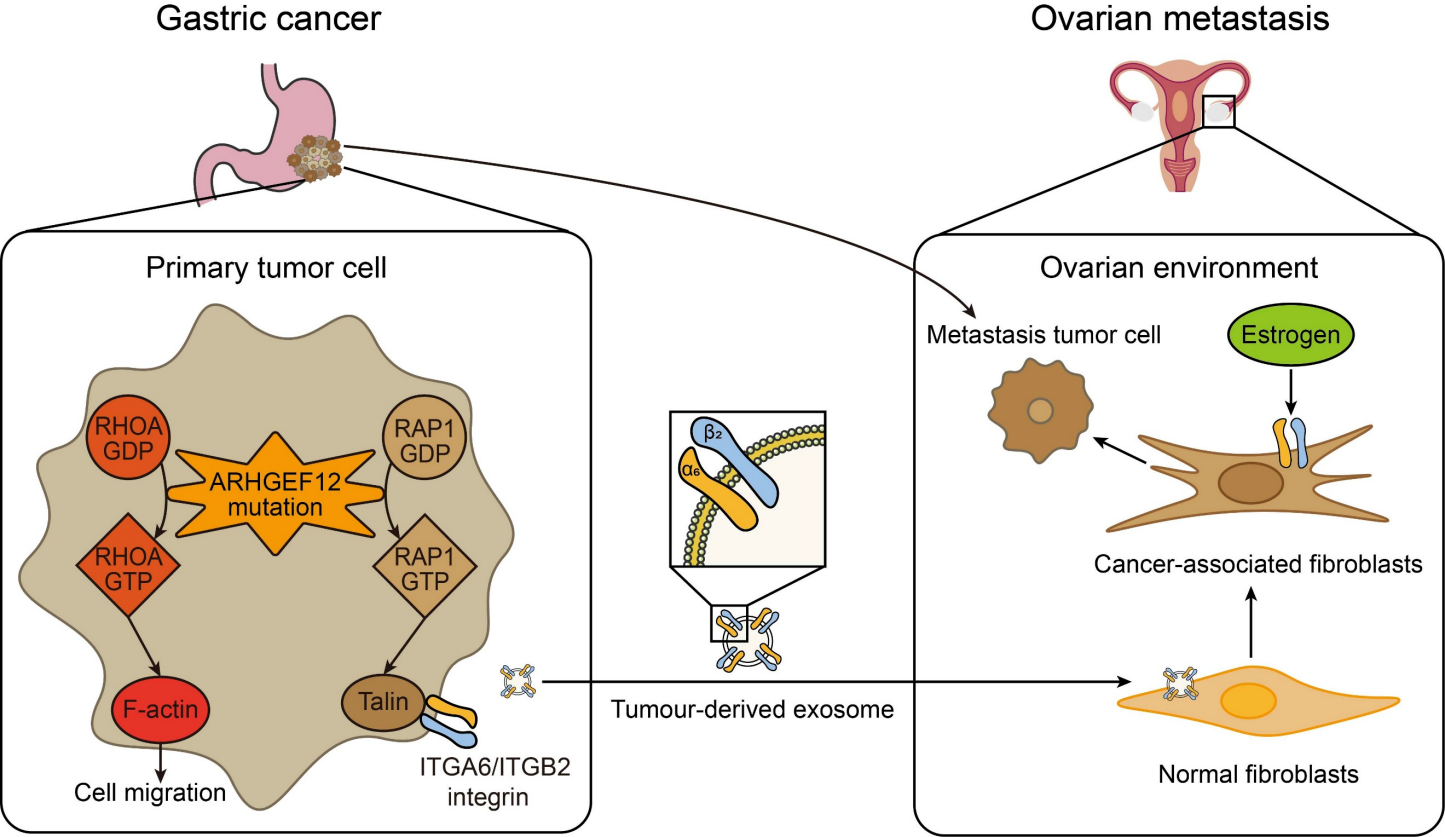
**A working model of *ARHGEF12* mutation promotes gastric cancer ovarian metastasis.** In gastric cancer cells, *ARHGEF12* mutation activates GTPases, including RhoA and Rap1A. RhoA-GTP promotes actin cytoskeleton organization, thereby enhancing migration ability of cancer cells. Rap1A-GTP upregulates integrin gene expression through Rap1 signaling pathway. Tumor-derived exosomes enriched with ITGA6 and ITGB2 proteins are predominantly uptaken by ovarian normal fibroblasts. These exosome-educated fibroblasts transform into cancer-associated fibroblasts, which promote metastasis tumor cell proliferation, thereby initiating the early stage of pre-metastatic niche formation.

The early diagnosis and effective treatment of gastric cancer (GC) with ovarian metastasis (OM) remain challenging. In this study, the genomic mutations of GC with OM were systematically evaluated by whole exome sequencing (WES). We identified seven specific mutated genes as early predisposition targets for GC with OM. Subsequent in-depth functional assays revealed that the E620K mutation of *ARHGEF12* as a gain-function mutation promotes OM of GC through tumor-derived ITGA6**-**high exosomes. These exosome-educated ovarian fibroblasts transform into cancer-associated fibroblasts (CAF), thereby initiating the early stage of pre-metastatic niche formation (Figure 9).

To the best of our knowledge, mutations of *FLCN*, *DNAJC13*, *DSC3*, *SLC9A3*, *ADGRV1*,* SCAPER* and *ARHGEF12* are first identified to be associated with OM of GC. Folliculin (*FLCN*) mutations have been linked to Birt-Hogg-Dubé syndrome 54-56. Mutations of DnaJ Heat Shock Protein Family Member C13 (*DNAJC13*) are associated with Parkinson's disease (PD) 57-60. A homozygous nonsense mutation in Desmocollin 3 (*DSC3*) gene resulted in skin fragility and hypotrichosis 61. Solute carrier family 9 member A3 (*SLC9A3*) mutations cause Congenital Sodium Diarrhea (CSD) and cystic fibrosis 62-64. Adhesion G protein-coupled receptor V1 (*ADGRV1*) plays a crucial role in hair cell development, and *ADGRV1* mutations are associated with Usher syndrome (USH) 65-67. Mutations in the gene S phase Cyclin A-Associated Protein residing in the Endoplasmic Reticulum (*SCAPER*) are associated with retinitis pigmentosa (RP) and intellectual disability (ID) 68-70. Our study not only identifies these mutated genes as predictors of GC with OM but also reveals their novel functions in cancer.

We also identified that recurrent mutations of *ARHGEF12* are associated with poor prognosis in GC. The fusion of *ARHGEF12* with histone-lysine N-methyltransferase 2A gene (*KMT2A*) has been reported in leukemic patients 22, 23, and *ARHGEF12* mutations have also been found in metastatic prostate cancer 71. As we know, we are the first to identify recurrent mutations of *ARHGEF12* in GC with OM and demonstrate that *ARHGEF12* mutations are recurrent in both GC and ovarian cancer patients. Notably, these recurrent mutations are associated with poor prognosis in GC. Moreover, high expression of ARHGEF12 is a prognostic factor for GC. ARHGEF12 is highly expressed in OM compared to matched primary tumors (PT). *ARHGEF12* depletion reduced tumor growth, whereas overexpression of *ARHGEF12* promoted OM of GC.

Although *ARHGEF12* mutations have been found in several cancers 22, 23, 71, the functions of these mutations remain largely unclear. In this study, we performed functional assays and identified E620K mutation of ARHGEF12 as a gain-of-function mutation. Re-expression of the E620K mutant markedly enhanced GC cell migration, invasion and colony formation, and significantly increased metastatic lung nodule formation and peritoneal metastasis in vivo through the activation of Rap1 signaling pathway.

OM of GCs can occur through multiple mechanisms, including lymph node metastasis, hematogenous metastasis, and seeding metastasis 3. Exosomes are small membrane vesicles (30-100 nm) secreted by tumor cells, have recently been recognized as 'primers' of the metastatic niche 42-44. Recently, a study on GCs with OM indicated that estrogen receptor positive (ER^+^) ovarian fibroblasts secrete midkine (MDK) under estrogen influence, which drives ovarian engraftment and metastasis of low-density lipoprotein receptor-related protein 1 positive (LRP1^+^) GC cells 14. Here, we demonstrate that GC tumor-derived ITGA6-high exosomes are preferentially uptaken by (ER^+^) ovarian fibroblasts and those exosome-educated fibroblasts exhibit CAF phenotypes, thereby initiating the early stages of pre-metastatic niche formation of OM.

Finally, our results showed that* ARHGEF12* overexpression induced chemoresistance to docetaxel but had no significant effect on sensitivity to 5-FU or oxaliplatin. Mechanistically, 5-FU blocks DNA replication, leading to S-phase cell cycle arrest 72. Oxaliplatin forms DNA crosslinks that induce structural DNA damage and activate apoptosis 73. Docetaxel binds to β-tubulin, stabilizing microtubule polymerization and preventing disassembly, which disrupts mitosis 74. Notably, ARHGEF12 is known to regulate cytoskeleton dynamics and cell polarization 19. Thus, we hypothesize that the observed docetaxel-specific chemoresistance arises from ARHGEF12-mediated modulation of cytoskeletal dynamics, which may interfere with the pharmacological mechanism of docetaxel.

## Conclusion

Overall, our study provides new insights into the genomic mutation profiling of GC with OM and identifies novel predictors. We also demonstrate that *ARHGEF12* mutation may contribute to OM of GC via tumor-derived exosomes containing specific integrins. These findings may advance our understanding of the metastatic process and promote early diagnosis and therapeutic intervention in GC with OM.

## Supplementary Material

Supplementary figures and tables.
