# Supplementary material for: Comprehensive exome profiling identifies ARHGEF12 mutation as a driver in gastric cancer with ovarian metastasis
Source: Theranostics. 2025 Jul 24;15(16):8202–21. doi: 10.7150/thno.113382 (PMC12374580; doi:10.7150/thno.113382)
Supplement: Supplementary file 1 — Supplementary figures and tables. [file thnov15p8202s1.pdf]

Supplementary Information

Comprehensive exome profiling identifies *ARHGEF12* mutation as a driver in gastric cancer with ovarian metastasis

Mingda Zhang, Guoyu Chen, Xiaolin Lin, Yingwen Zhang, Longyu Shi, Shanshan Li, Yanxin Li, Xiuying Xiao, and Haizhong Feng

Correspondence: Haizhong Feng, E-mail: fenghaizhong@sjtu.edu.cn, Xiuying Xiao, E-mail: xiaoxiuying2002@163.com, or Yanxin Li, E-mail: liyanxin@scmc.com.cn

This Word file contains the following:

Supplemental Table S1-S3

Supplemental Figure S1-S8

Table S1 Clinical and pathological features of gastric cancer (GC) patients

Patient ID	Primary Tumor	Ovarian metastases	Sex	Age	Menstrual history (Pre = 1, Post = 2)	Pathological type (Signet ring cell = 1, Non-signet ring cell = 2)	Differentiation (Poor = 1, Well/moderate = 2)	Chronology (Synchronous = 1, Metachronous = 2)	Laterality (Bilateral = 1, Unilateral = 2)	Metastasis site	Foci resection (Radical = 1, Palliative = 2)
P1	PT1	OM1	F	28	1	1	1	1	1	Ovary, peritoneum	2
P2	PT2	OM2	F	41	1	2	1	1	1	Ovary	1
P3	PT3	OM3	F	40	1	2	1	2	1	Ovary	1
P4	PT4	OM4	F	34	1	2	1	2	1	Ovary, peritoneum	2
P5	PT5	OM5	F	39	1	2	1	1	1	Ovary	1
P6	PT6	OM6	F	40	1	1	1	1	1	Ovary, peritoneum	2
P7	PT7	OM7	F	46	1	1	1	1	1	Ovary	2
P8	PT8	OM8	F	54	2	1	1	1	1	Ovary	2
P9	PT9		F	64	2	2	2			Liver, peritoneum	2
P10	PT10		F	38	1	2	1			Peritoneum	2
P11	PT11		F	45	1	1	1			Peritoneum	2
P12	PT12		F	20	1	1	1			Peritoneum	2
P13	PT13		F	64	2	1	1			Peritoneum	2
P14	PT14		F	51	2	1	1			Peritoneum	2

Table S2 Sequences for shRNA and sgRNA targeting *ARHGEF12*

	Sequence
shRNA #1	5'- GCGAGTATCCAGAGAAGGAAT -3'
shRNA #2	5'- GCGTTGCGTAATCATCCAGAA -3'
sgRNA #1	5'- ACTTTCTGCTTCTTATCTGT -3'
sgRNA #2	5'- ATCATCCAGAAAGATGACAA -3'
sgRNA #3	5'- GAAGACAAAGTCTAGTTCAG -3'
sgRNA #4	5'- GGATTTGGGCTGACGGTCAG -3'

Table S3 Primers for qRT-PCR assays

Gene	Sequence
ITGA1	5'- CACTGTTGTTCTACGCTGCTG -3' and<br>5'- ACGGAGAACCAATAAGCACCC -3'
ITGA2	5'- GTGGCTTTCCTGAGAACCGA -3' and<br>5'- GATCAAGCCGAGGCTCATGT -3'
ITGA3	5'- GTGGCTTCACCCAGAACACT -3' and<br>5'- TGAAGCTGCCTACCTGCATC -3'
ITGA5	5'- GTCGGGGGCTTCAACTTAGA -3' and<br>5'- GGCTGGTATTAGCCTTGGGT -3'
ITGA6	5'- TTGGAGCCCCGGGTACTTAT -3' and<br>5'- GCAGGAACAGGAACGAGACT -3'
ITGA7	5'- TACAACCAGAATTCAGAGGCAGGC -3' and<br>5'- ATCGGTGTGCACAGGTTTCCT -3'
ITGA10	5'- GTGAGCTCTGCCCATTGGAT -3' and<br>5'- GTTCCTTGGAGGGTCAGCAA -3'
ITGAX	5'- CTCCTGTTCACAGCCTTAGCA -3' and<br>5'- TGCTGTAGCCACACTGGTAG -3'
ITGAM	5'- GCTTTGGTGGCTTCCTTGTG -3' and<br>5'- CCCCTTGCGTTCTCTTGGAA -3'

ITGAV	5’- TCCGAAGCTCAGCCCTCTT -3’ and<br>5’- GGAAAAGCCATCGCCGAAGT -3’
ITGB1	5’- ATGTGTCAGACCTGCCTTGG -3’ and<br>5’- GCTGGGGTAATTTGTCCCGA -3’
ITGB2	5’- GTACTGCGAGTGTGACACCA -3’ and<br>5’- GCCACGACCACTACACTCAA -3’
ITGB4	5’- CGAGGTAGGTCCAGGACGG -3’ and<br>5’- TTTGCCAAGGTCCCAGAGAG -3’
ITGB5	5’- GTCTCGGAGCCCAAGTCG -3’ and<br>5’- GGCCGGGACTCCTAGTGT -3’
ITGB8	5’- GCGAAAAGGACAAGGGCAC -3’ and<br>5’- GGAGCGCCCTAGAAGCAC -3’

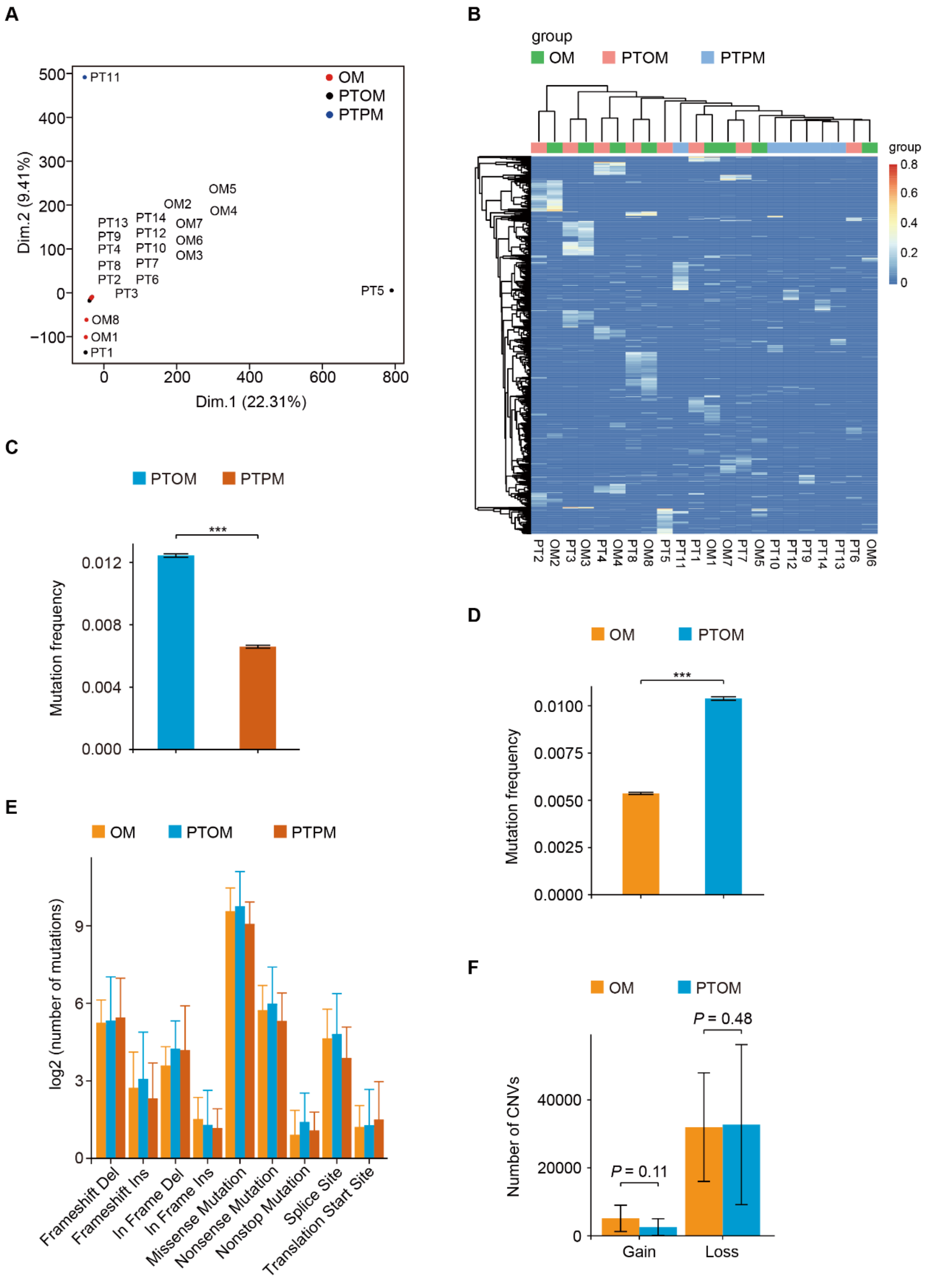

Figure S1. Comparison of mutation frequencies in gastric cancer (GC) with ovarian metastasis (OM).

(A) Principal-component analysis (PCA) of fourteen primary tumors (PT) and eight

ovarian metastases (OM). Each point corresponds to an individual sample, plotted according to its scores on the first two principal components PC1 and PC2. PTOM, primary tumors with ovarian metastasis. PTPM, primary tumors with peritoneal metastasis.

(B) Heatmap of mutation frequencies in different samples.

(C) Comparison of mutation frequencies between PTOM and PTPM.

(D) Comparison of mutation frequencies between OM and PTOM.

(E) Comparison of ratios of mutations across sample groups.

(F) Comparison of gain (>4) or loss (<2) copy number variations (CNVs) between PTOM and OM.

Data were presented as mean ± 95%CI. ***$P < 0.001$, by two-tailed Student's *t*-test.

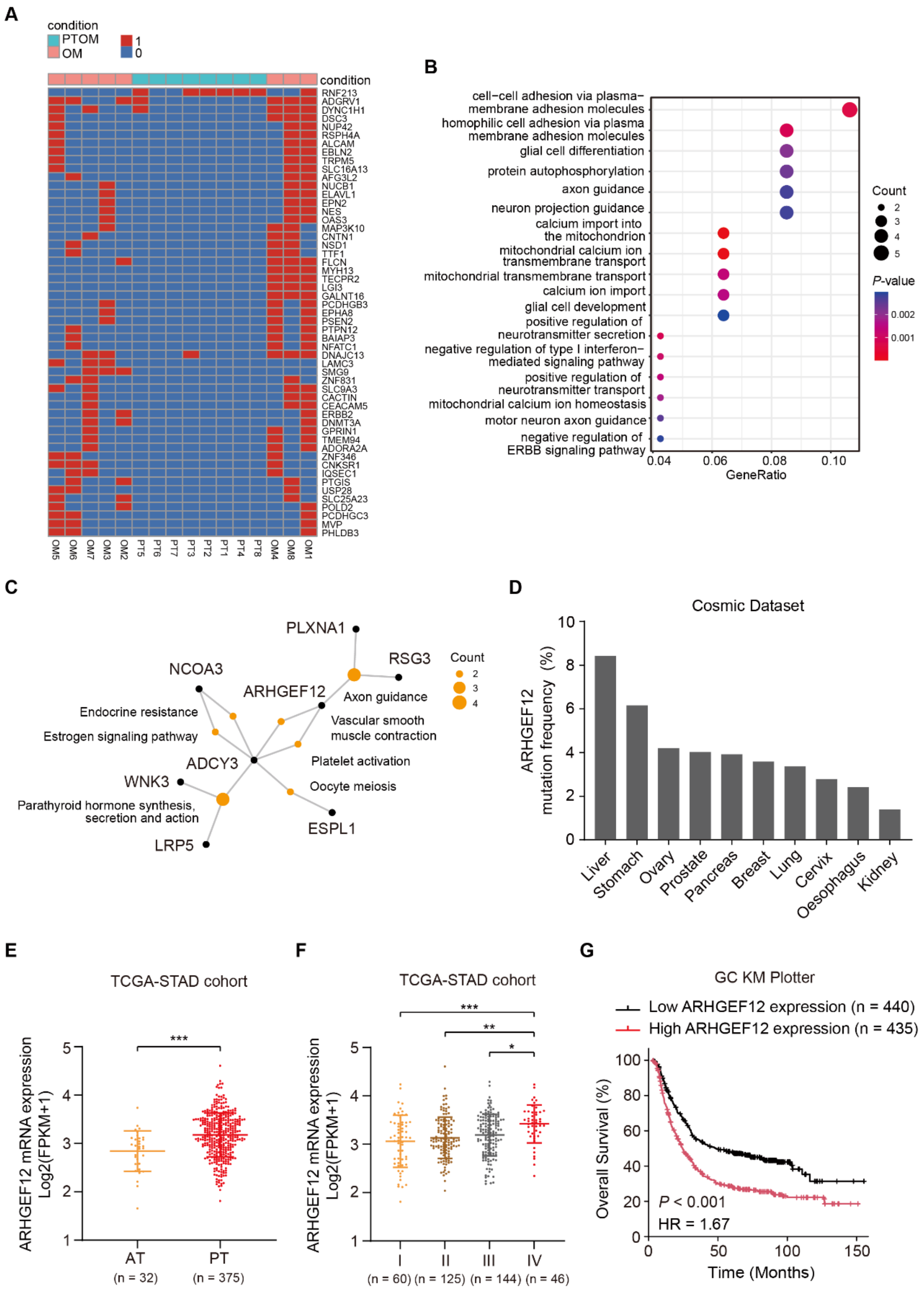

Figure S2. ARHGEF12 is a prognostic factor for GC.

(A) Heatmap of specifically gain- or loss-of-function mutations in OM compared to PTOM.

(B) Gene Ontology (GO) enrichment analysis of de novo mutations in OM.

(C) Schematic diagram of KEGG pathways and associated genes.

(D) Mutation frequencies of *ARHGEF12* in different cancer types from the Cosmic dataset. Gastric cancer exhibited the second highest *ARHGEF12* mutation frequency (6.16%, 100 out of 1623) among various cancer types.

(E) Comparison of *ARHGEF12* mRNA levels between PT and adjacent tissues (AT) from TCGA cohort.

(F) Comparison of *ARHGEF12* mRNA levels between different stages in GC patients from TCGA cohort.

(G) High *ARHGEF12* expression correlates with poor overall survival (OS) in GC specimens in the Kaplan-Meier plotter dataset.

Data were presented as mean ± SEM. *$P < 0.05$, **$P < 0.01$, ***$P < 0.001$, by two-tailed Student's *t*-test or log-rank test.

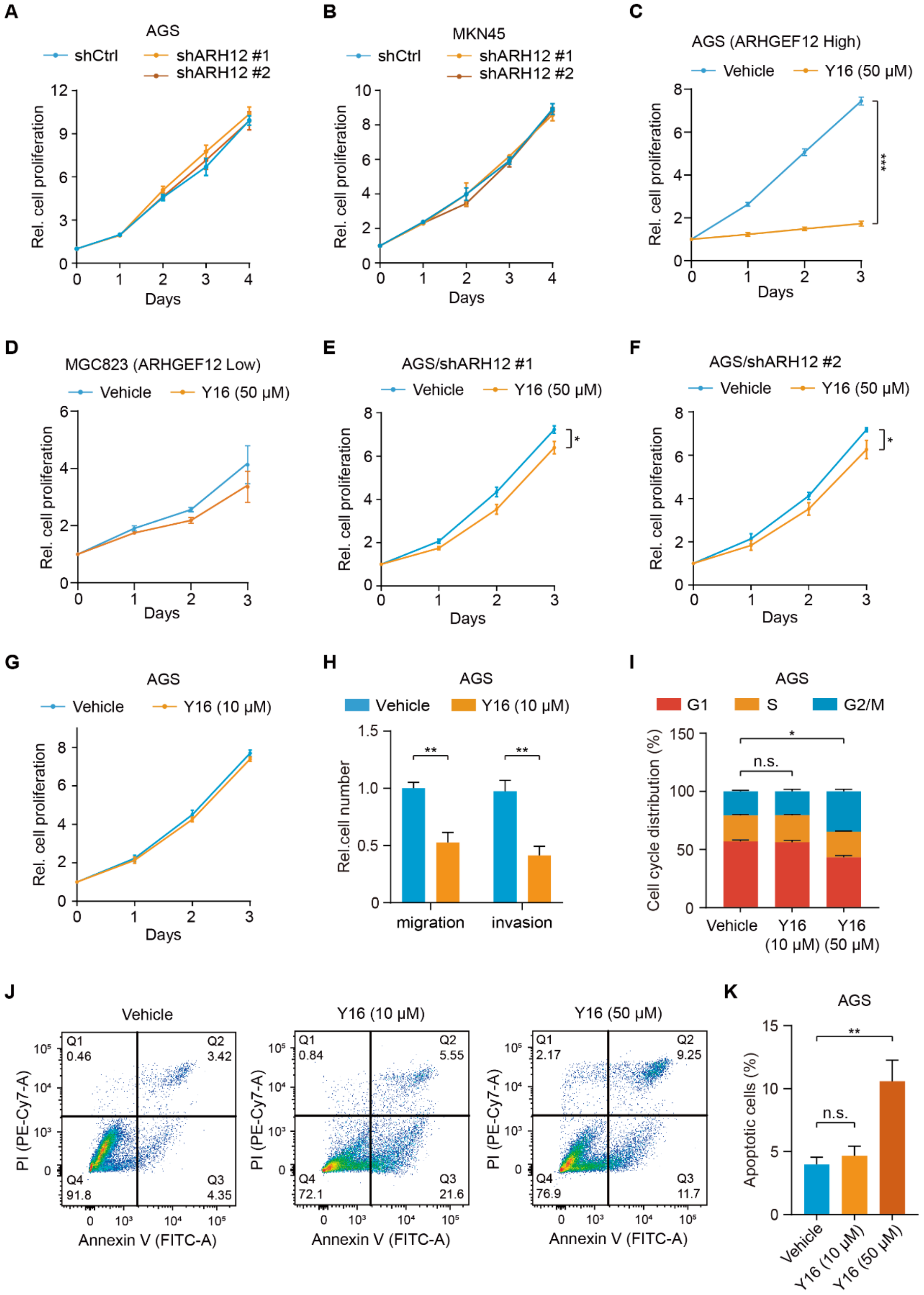

Figure S3. Knockdown of *ARHGEF12* inhibits metastasis of GC cells.

(A-B) Effects of *ARHGEF12* knockdown (KD) on AGS (A) and MKN45 (B) cells proliferation.

(C-D) Cell proliferation analysis of AGS cells (high ARHGEF12 expression, C) or MGC823 cells (low ARHGEF12 expression, D). Cells were treated with 50 μM Y16 for 0, 24, 48 or 72 h, respectively.

(E-F) Cell proliferation analysis of AGS/shARHGEF12 cells. Cells were treated with 50 μM Y16 for 0, 24, 48 or 72 h, respectively.

(G) Cell proliferation analysis of AGS cells. Cells were treated with 10 μM Y16 for 0, 24, 48 or 72 h, respectively.

(H) Cell migration and invasion analysis. Cells were pre-treated with or without 10 μM Y16 for 48 h and then subjected to cell migration and invasion assays.

(I) Quantitative analysis of cell cycle distributions in AGS cells treated with 10 or 50 μM Y16 for 24 h, respectively.

(J) Representative flow cytometry plots of AGS cells treated with 10 or 50 μM Y16 for 24 h, respectively.

(K) Quantification of apoptotic cells in (J).

Data are representative of three independent experiments with similar results. Data were presented as mean ± SEM. n.s., no significant, **P* < 0.05, ***P* < 0.01, by two-tailed Student's *t*-test or one-way ANOVA analysis.

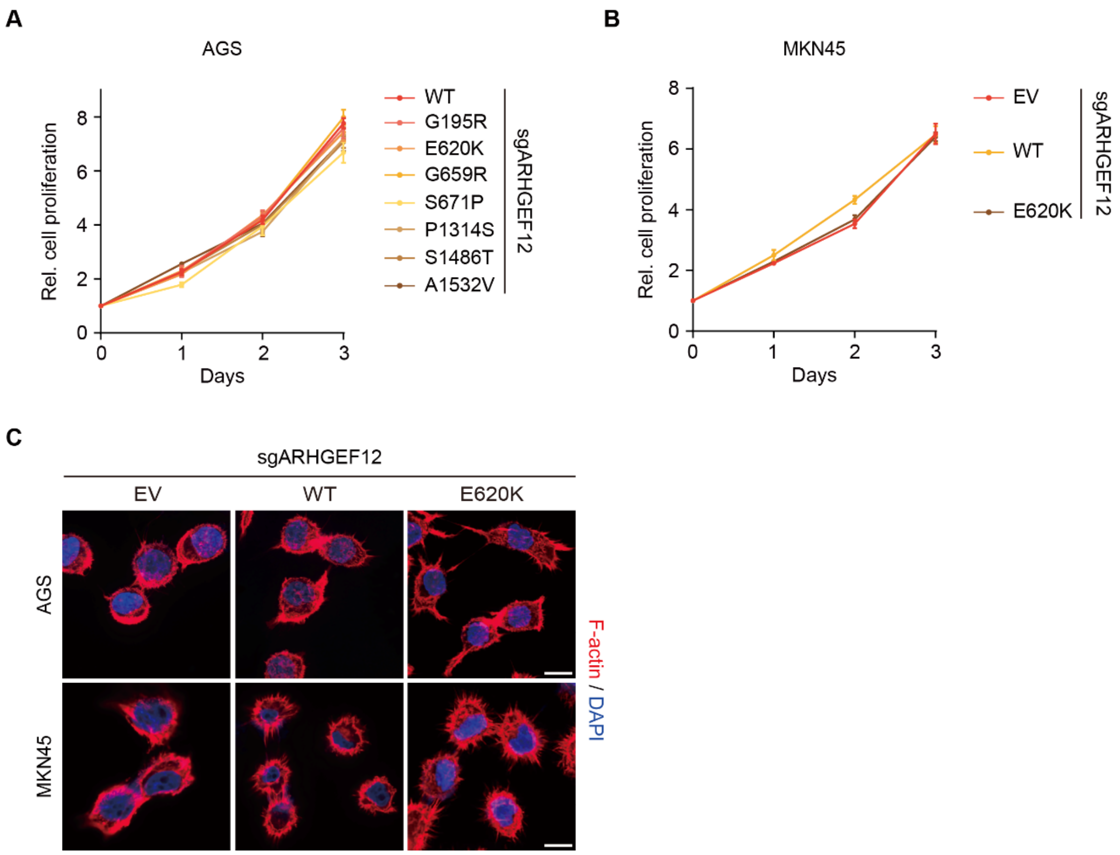

Figure S4. E620K mutation of *ARHGEF12* promotes OM in GC.

(A-B) Effects of *ARHGEF12* mutation on AGS (A) and MKN45 (B) cells proliferation. Empty vector (EV), wild-type (WT) *ARHGEF12* or *ARHGEF12* mutants were re-expressed in *ARHGEF12* knockout (sgARHGEF12) cells.

(C) Representative immunofluorescence (IF) images of *ARHGEF12* knockout AGS and MKN45 cells, with re-expression of EV, WT *ARHGEF12* and *ARHGEF12* E620K mutant. Red: F-actin, Blue: DAPI. Scale bar, 10 μm.

Data are representative of three independent experiments with similar results.

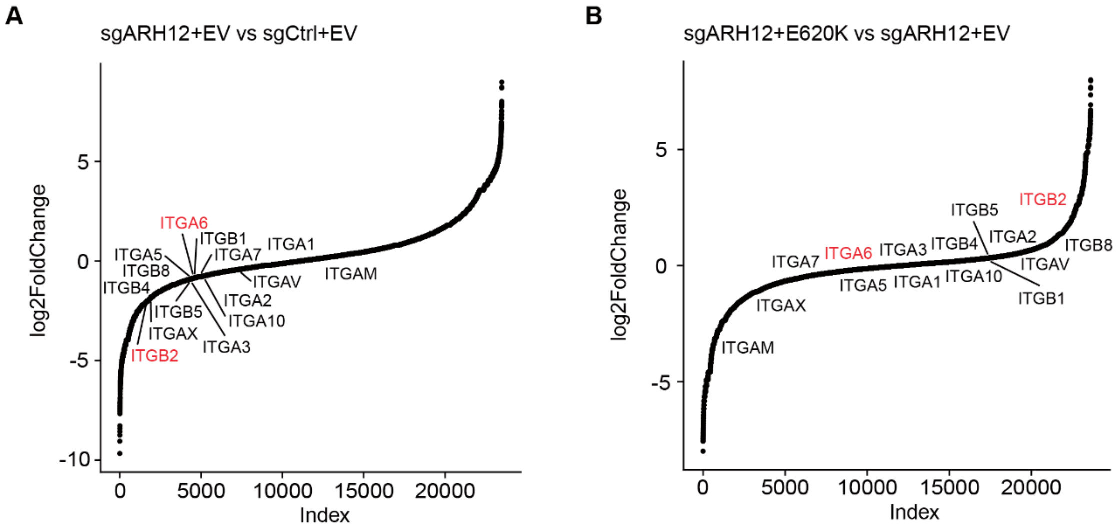

Figure S5. *ARHGEF12* E620K mutation upregulates integrin gene expression.

(A-B) Genes ranked by fold change following ARHGEF12 KO (A) or re-expression of the E620K mutant (B). All integrin genes are labeled.

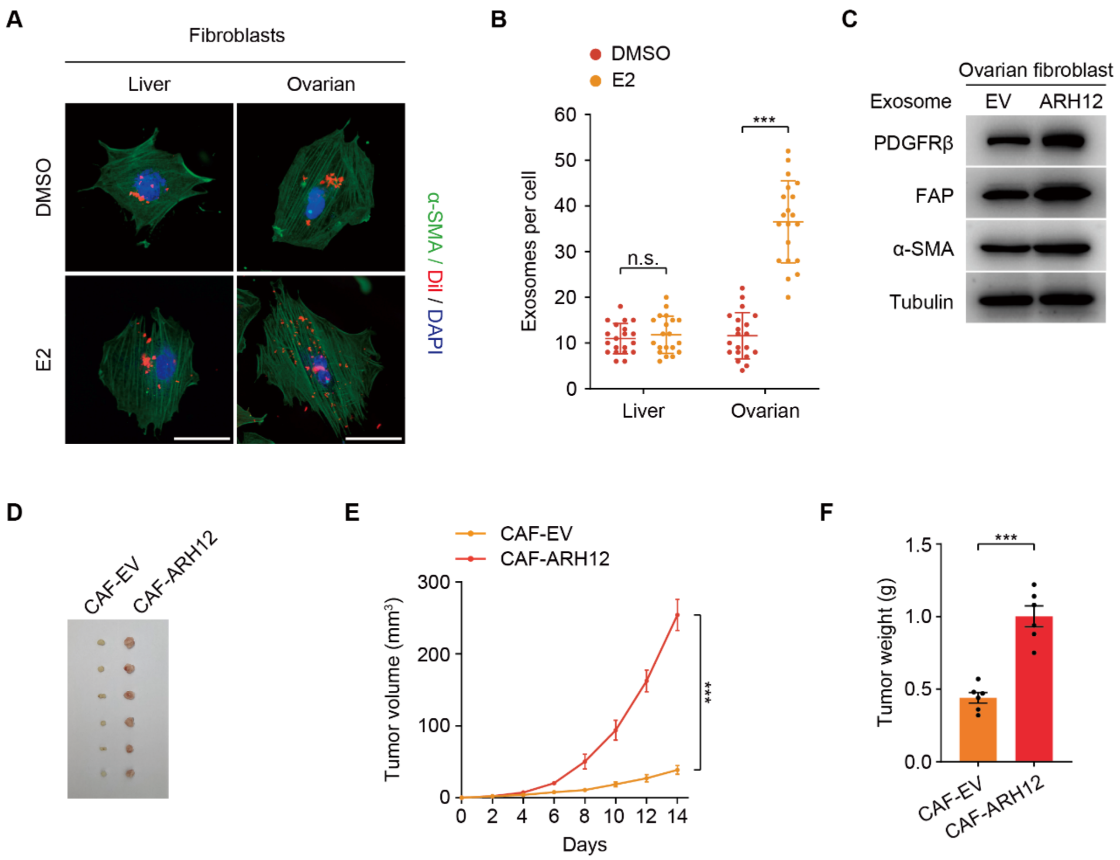

Figure S6. Tumor-derived ITGA6-high exosomes are predominantly uptaken by ovarian fibroblasts.

(A) IF staining of mouse liver and ovarian fibroblasts stimulated with estradiol (E2) and co-cultured with exosomes from MFC cells. Fibroblasts were stained with α-SMA. Scale bars, 50 μm.

(B) Quantification of mouse ovarian fibroblasts uptake of tumor-derived exosomes in (A). Data shown are from 20 cells counted per condition.

(C) WB of the expression of cancer-associated fibroblasts (CAF) markers α-SMA, FAP and PDGFRβ in ovarian fibroblasts co-cultured with tumor-derived exosomes.

(D) Representative image of tumor masses. MFC cells mixed with exosome-educated fibroblasts were implanted into animals (n = 6).

(E) Tumor growth curves of xenografts in (D).

(F) Quantification of tumor weights in (D).

Data are representative of three independent experiments with similar results. Data were presented as mean ± SEM. n.s., no significant, $^{***}P < 0.001$, by two-tailed Student's *t*-test.

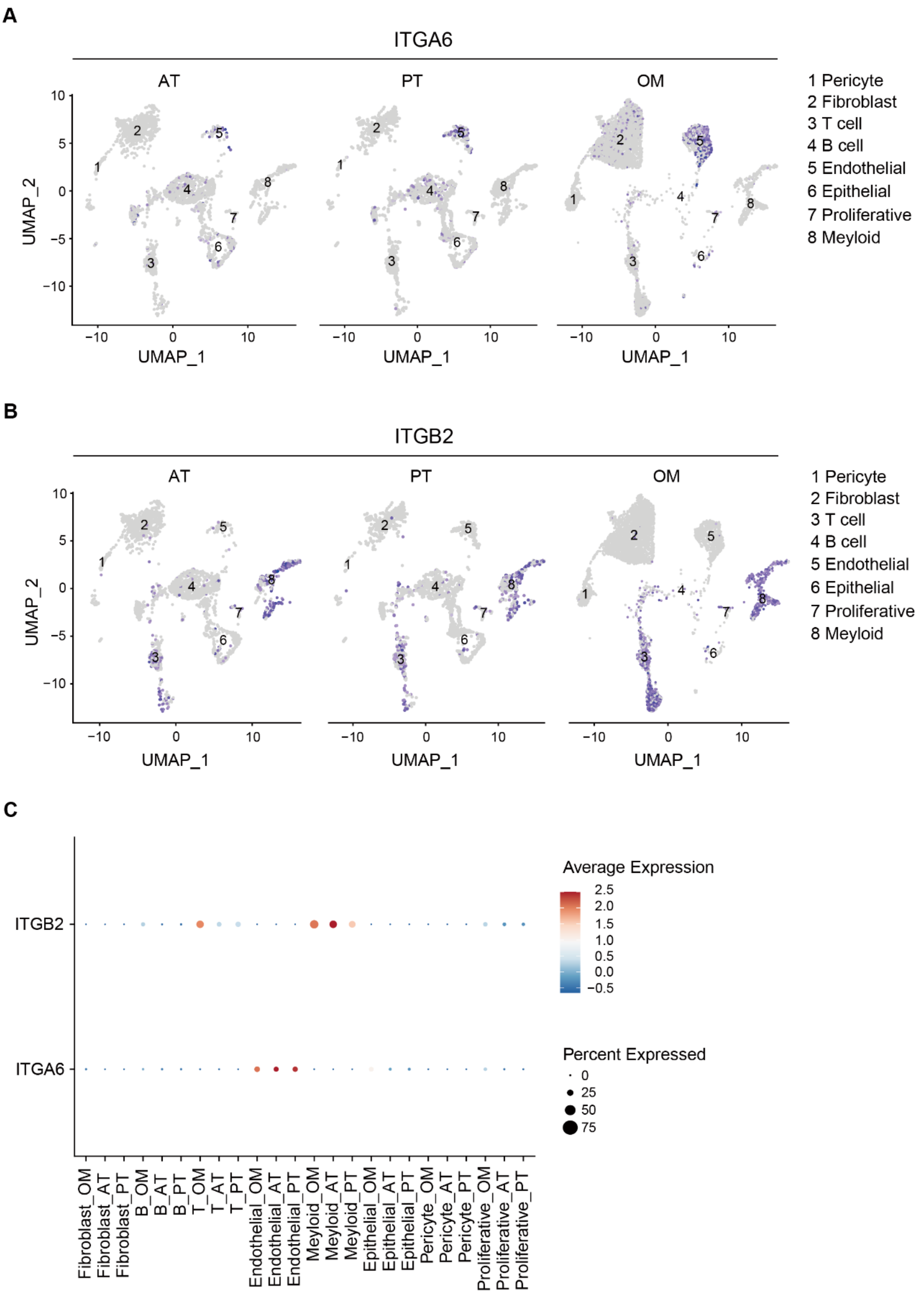

Figure S7. Single-cell RNA sequencing reveals *ITGA6* and *ITGB2* expression in ovarian microenvironment.

(A-B) Single-cell RNA sequencing analysis of matched adjacent tissue (AT), primary

tumor (PT) and ovarian metastases (OM) from 2 GC patients with OM. Uniform manifold approximation and projection (UMAP) visualization of *ITGA6* (A) and *ITGB2* (B) expression across different samples. The intensity of color indicated the average expression levels.

(C) Dot plot showing *ITGA6* and *ITGB2* expressions in different cell subtypes. The dot sizes indicate the percentage of cells in each cluster expressing the gene and the colors indicate average expression levels.

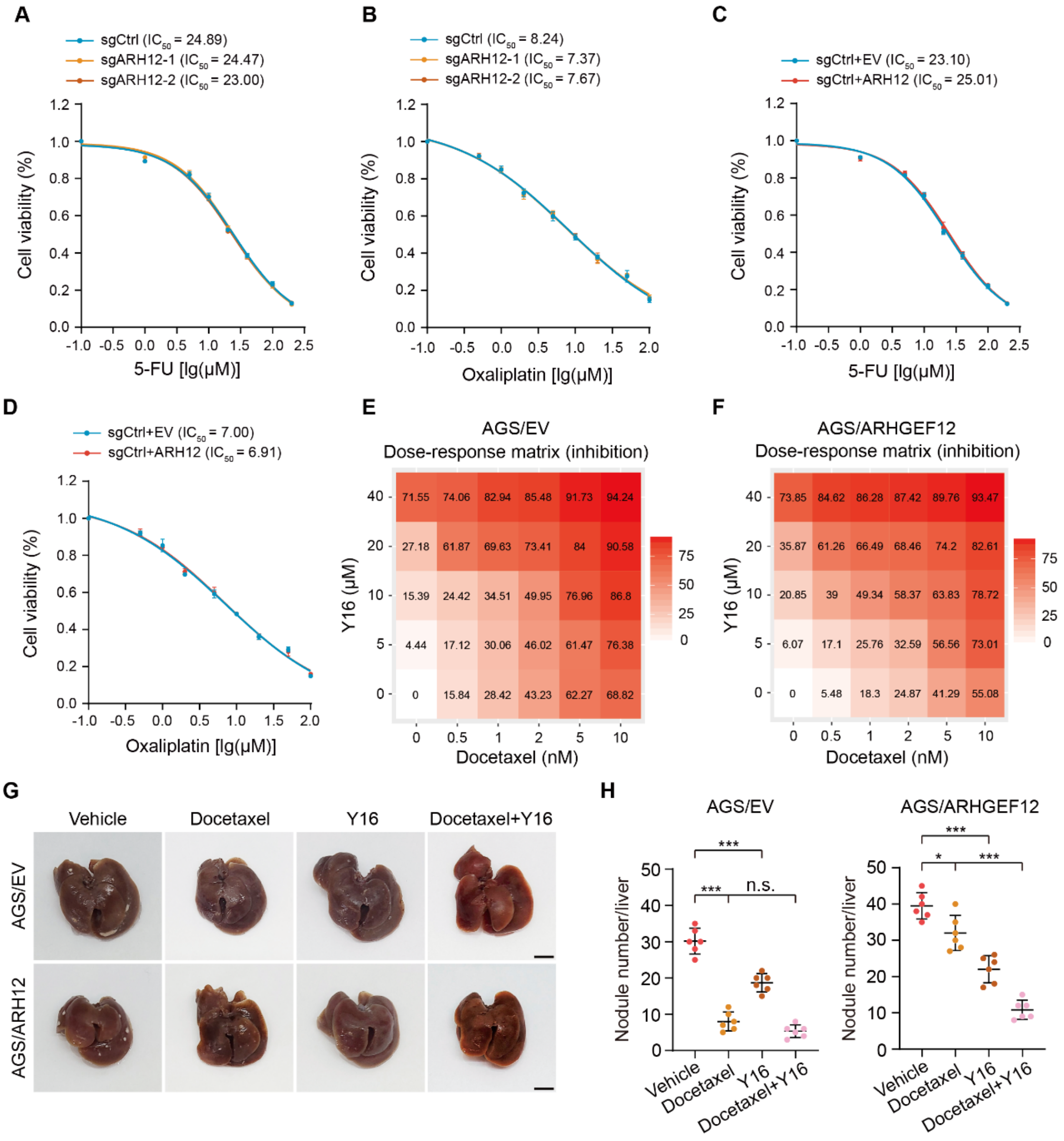

Figure S8. Targeting ARHGEF12 with Y16 synergizes with docetaxel to suppress OM.

(A-B) Cell viability analysis. AGS cells with sgARHGEF12 or sgCtrl were treated with 5-FU (A) or oxaliplatin (B) for 48 h.

(C-D) Cell viability analysis. AGS cells with or without ectopic expression of *ARHGEF12* (AGS/ARHGEF12) were treated with 5-FU (C) or oxaliplatin (D) for 48 h.

(E-F) Dose-response matrix. AGS/EV (E) or AGS/ARHGEF12 (F) cells were treated with docetaxel or Y16 alone or together. The inhibition rate was shown in matrix.

(G) Representative bright-field images of the animals metastatic-burden livers with docetaxel or/and Y16 treatment. Scale bar, 600 μm.

(H) The number of macroscopic lesions on the liver surfaces in (G) was quantified by necropsy.

Data are representative of three independent experiments with similar results. Data were presented as mean ± SEM. n.s., no significant, *$P < 0.05$, ***$P < 0.001$, by two-tailed Student's *t*-test.
